# *De novo* cyclic peptides allow visualisation of the monomeric and functional amyloid conformations of the kinase RIPK3

**DOI:** 10.1042/BCJ20250283

**Published:** 2026-02-04

**Authors:** Jessica A. Buchanan, Olivia Lavidis, Nicholas Shields, Katriona Harrison, Huy T. Nguyen, Richard J. Payne, Toby Passioura, Megan Steain, Margaret Sunde

**Affiliations:** 1 School of Medical Sciences and Sydney Infectious Diseases, The University of Sydney, Sydney, NSW 2006, Australia; 2 School of Chemistry and Australian Research Council Centre of Excellence for Innovations in Peptide and Protein Science, The University of Sydney, Sydney, NSW 2006, Australia; 3 Sydney Analytical Core Research Facility, The University of Sydney, Sydney, NSW 2006, Australia

**Keywords:** amyloid, necroptosis, peptides, RIPK3, self-assembly

## Abstract

Receptor interacting protein kinase 3 (RIPK3) is a central regulator of necroptosis and a key mediator of inflammatory signalling. Its function is orchestrated through RIP homotypic interaction motif (RHIM)-dependent interactions with other RHIM-containing adapter proteins, forming amyloid-structured necrosomes that trigger kinase activation and subsequently lead to immunogenic cell lysis. Necroptosis eliminates infected or damaged cells and provokes a strong inflammatory response. Dysregulated necroptosis is implicated in chronic inflammatory diseases and associated with ischaemic injury. Despite separate structural elucidation of the isolated RIPK3 kinase domain and the amyloid fibrils formed by the RIPK3 RHIM-containing region, visualising full-length human RIPK3 in live cells remains challenging due to a lack of specific tools. To address this, we employed random non-standard peptide integrated discovery (RaPID) mRNA display to identify cyclic peptides that bind the RHIM-containing region of RIPK3 during its assembly into amyloid fibrils. Three peptides were selected for characterisation and demonstrate utility in visualising RIPK3 in human cells. These peptides represent promising tools for probing RIPK3 localisation and modulating its function.

## Introduction

Receptor interacting protein kinase 3 (RIPK3) is a crucial co-ordinating regulator of the lytic programmed cell death pathway known as necroptosis [1,2]. It also plays key roles in NF-κB signalling, inflammasome activation and kinase-independent apoptosis [1,3–5]. RIPK3 contains an 18–20-residue RIP homotypic interaction motif (RHIM), through which it forms complexes called necrosomes with other RHIM-containing adapter proteins: RIPK1, TIR-domain-containing adapter-inducing interferon-β (TRIF) and Z-DNA-binding protein 1 (ZBP1) [6]. Necroptosis can be triggered by inflammatory mediators, the detection of pathogen-associated molecular patterns from fungal and bacterial infection, or through intracellular sensing of cellular or viral Z-form DNA or RNA [7,8].

**Figure 1:**
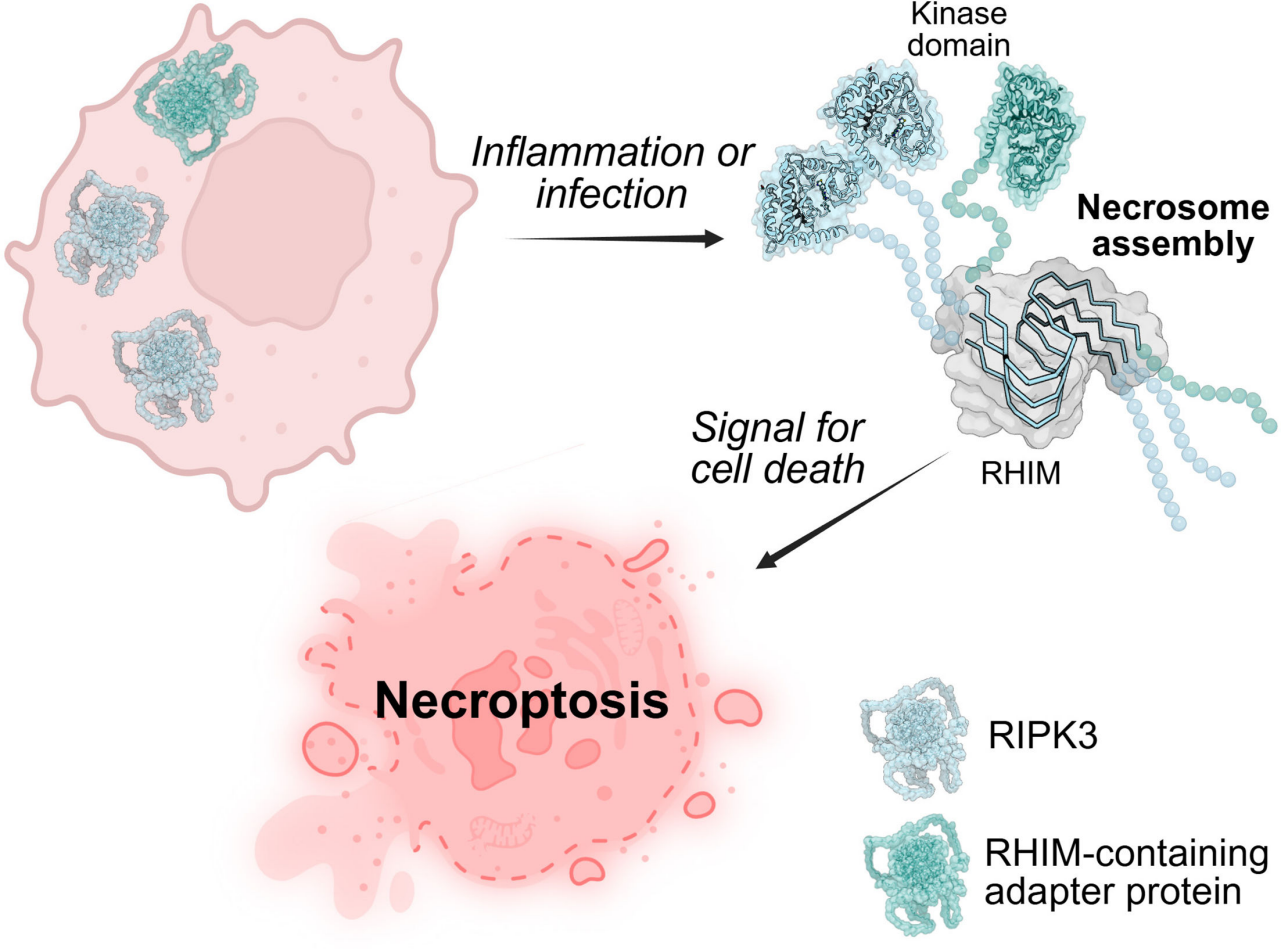
Necrosome formation. Cytosolic, monomeric RIPK3 and RHIM-containing adapter proteins are activated by inflammation or fungal, bacterial or viral infection. RIPK3 and adapter proteins form the amyoid necrosome structure via their RHIMs, leaving kinase domains available along the fibril surface. Necrosome formation triggers downstream signalling for lytic, inflammatory cell death. Figure generated using BioRender.

The role of RIPK3 as a hub within both protective inflammatory signalling and in cell death pathways makes it of key interest in pathological conditions associated with unwanted necroptosis, as well as holding potential for therapeutic induction of necroptosis, for example, in cancer treatment [5]. RHIM sequences also occur in some viral proteins which inhibit host defence upon infection by incorporating into the necrosome complex via their RHIM and preventing necroptosis signal propagation [9–13]. Necrosomes have an amyloid cross-β fibril architecture, formed when the RHIMs from the constituent proteins interact to generate a β-sheet-rich fibril core that brings the adapter proteins into close contact with RIPK3 [14] (illustrated in Figure 1).

The amyloid fibril-forming RHIM sequences render RIPK3 and the adapter proteins challenging candidates for structural studies. The structures of the isolated kinase domains of human and murine RIPK3 have been determined by crystallography [15] and separately, the structures of the fibrils formed by the RHIM-containing regions of human and murine RIPK3 have been determined by cryoEM [16]. There is solution NMR evidence for a relatively structured ‘preRHIM’ region of ~20 residues immediately N-terminal of the RHIM [17]. However, little is known about the cellular location and interactions of full-length RIPK3 before and following induction of necroptosis. Efforts to express and study full-length RIPK3 have been unsuccessful, and even the isolated kinase domain is relatively unstable [18]. Studies of human RIPK3 (hRIPK3) have been hindered by a lack of effective tools to visualise the protein within live cells. Multiple antibodies have been raised against hRIPK3, but these suffer from poor selectivity, and they are ineffective as reporters in immunofluorescence-based studies for monitoring hRIPK3 [19,20].

Cyclic peptides offer opportunities for effective binding of proteins such as hRIPK3 which self-assemble into amyloid fibrils with large, iterative and hydrophobic surfaces. Indeed, the relatively large binding surface area of cyclic peptides makes them more selective than small molecule probes for such targets [21]. Additionally, because cyclic peptides require a structured target to bind to, target selective binding can be achieved by cyclic peptides in cases where selective antibodies cannot be identified [22]. The random non-standard peptide integrated discovery (RaPID) system offers the combination of mRNA display with genetic code reprogramming, resulting in the ribosomal synthesis of expansive libraries containing > 10^12^ macrocyclic peptides [13].

Here, we report the application of RaPID to identify and select novel cyclic peptide binders with affinity for the 387–518 region of hRIPK3 (hRIPK3_387–518_), which is the RHIM-containing minimal region of human RIPK3 required for necrosome assembly [23]. The selection was applied during the process of amyloid fibril assembly. Three peptides were identified and their mode of binding to hRIPK3 characterised. Strikingly, these peptides were found to be capable of binding to hRIPK3 in both its monomeric and amyloid conformations. Additionally, these peptides are cell permeable and non-cytotoxic, allowing the visualisation of hRIPK3 within human cells and providing a starting point for the development of novel modulators of hRIPK3 activity.

## Results

### Identification of cyclic peptide hRIPK3 ligands using RaPID screening

**Figure 2:**
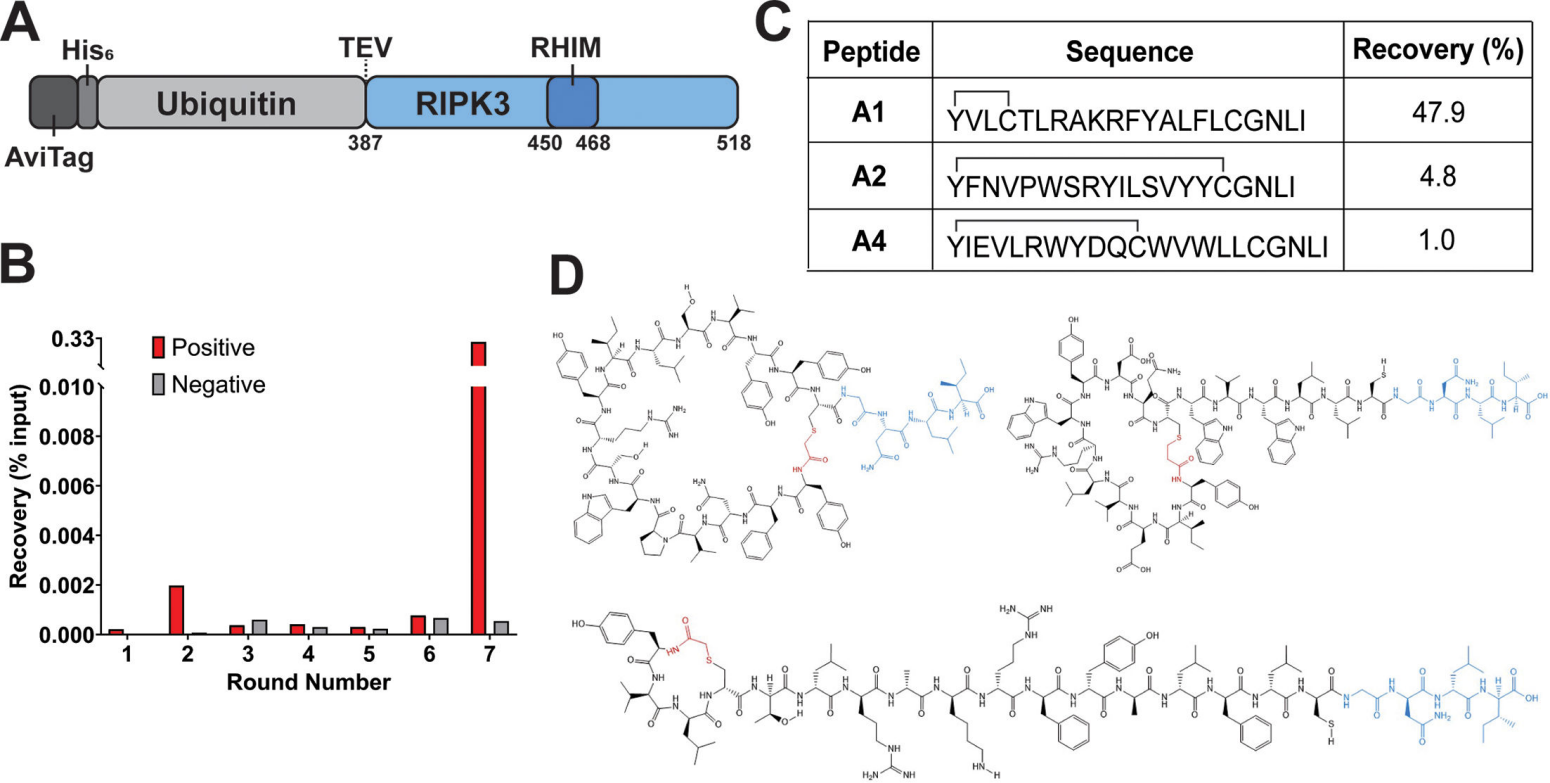
RaPID cyclic screening against BHUR3. (**A**) Schematic of BHUR3 construct used for RaPID screening. (**B**) Peptide recovery determined by RT-qPCR for positive and negative (counter) selection as a percentage of the total library input for each round of RaPID screening. Round 1 did not include a negative selection step. (**C**) Sequences and total percent recovery of peptides selected for characterisation. (**D**) Schematic of A1 (bottom), A2 (top left) and A4 (top right) cyclic peptides. GNLI linker shown in blue, cyclisation shown in red. Schematics created using ChemDraw.

The minimum necrosome-forming region hRIPK3_387–518_ was first expressed with an N-terminal AviTag for biotinylation to allow for protein immobilisation to streptavidin beads during RaPID screening. This construct also contained a hexahistidine (His_6_) tag for purification and a ubiquitin fusion partner for improved expression (Figure 2A). Complete biotinylation of this construct (henceforth referred to as BHUR3) was achieved via BirA ligase co-expression and biotin supplementation during protein expression. Fibril formation and fibrillar morphology were confirmed as equivalent to that of the non-biotinylated construct (HUR3) through monitoring of fibril formation kinetics using Thioflavin T (ThT), a widely used amyloid reporter fluorescent dye, and with transmission electron microscopy (Supplementary Figure S1).

For RaPID screening, a cyclic peptide library was produced via *in vitro* translation such that peptides consisted of an initiating *N*-chloroacetyl-ʟ-tyrosine residue followed by a random sequence of 5–15 residues and concluding with a cysteine residue and C-terminal linker sequence (Gly-Asn-Leu-Ile). Spontaneous peptide cyclisation occurred during screening via thioether bond formation between the N-terminal chloroacetyl moiety and the closest cysteine sulfhydryl group. Peptides remained linked to their encoding nucleic acid through ligation of a puromycin molecule to the library of mRNA prior to translation [13,24].

RaPID screening was performed against BHUR3 during amyloid assembly. Briefly, BHUR3 was diluted out of denaturing buffer into the cyclic peptide library and allowed to incubate for 20 minutes at room temperature with gentle agitation, conditions known to lead to amyloid assembly. Following this incubation, the protein was immobilised onto streptavidin-coated magnetic beads to recover binding peptides. Counter-selection was performed against biotinylated His_6_-ubiquitin (BHU) to remove non-hRIPK3 binding peptides from the library (see supporting information for full methodology). Enrichment was detected in the positive selection library after seven rounds of screening via RT-qPCR (Figure 2B). Strikingly, almost 50% of the selected peptides corresponded to a single sequence, peptide A1, with the next most highly represented peptides A2 and A4 at 4.8 and 1.0%, respectively (Figure 2C). These three peptides (A1, A2 and A4, Figure 2D) were therefore synthesised by Fmoc-SPPS (see supporting information for synthetic details) and taken forward for characterisation.

### hRIPK3-binding cyclic peptides required tetra-lysine tags to abrogate spontaneous self-assembly

**Figure 3:**
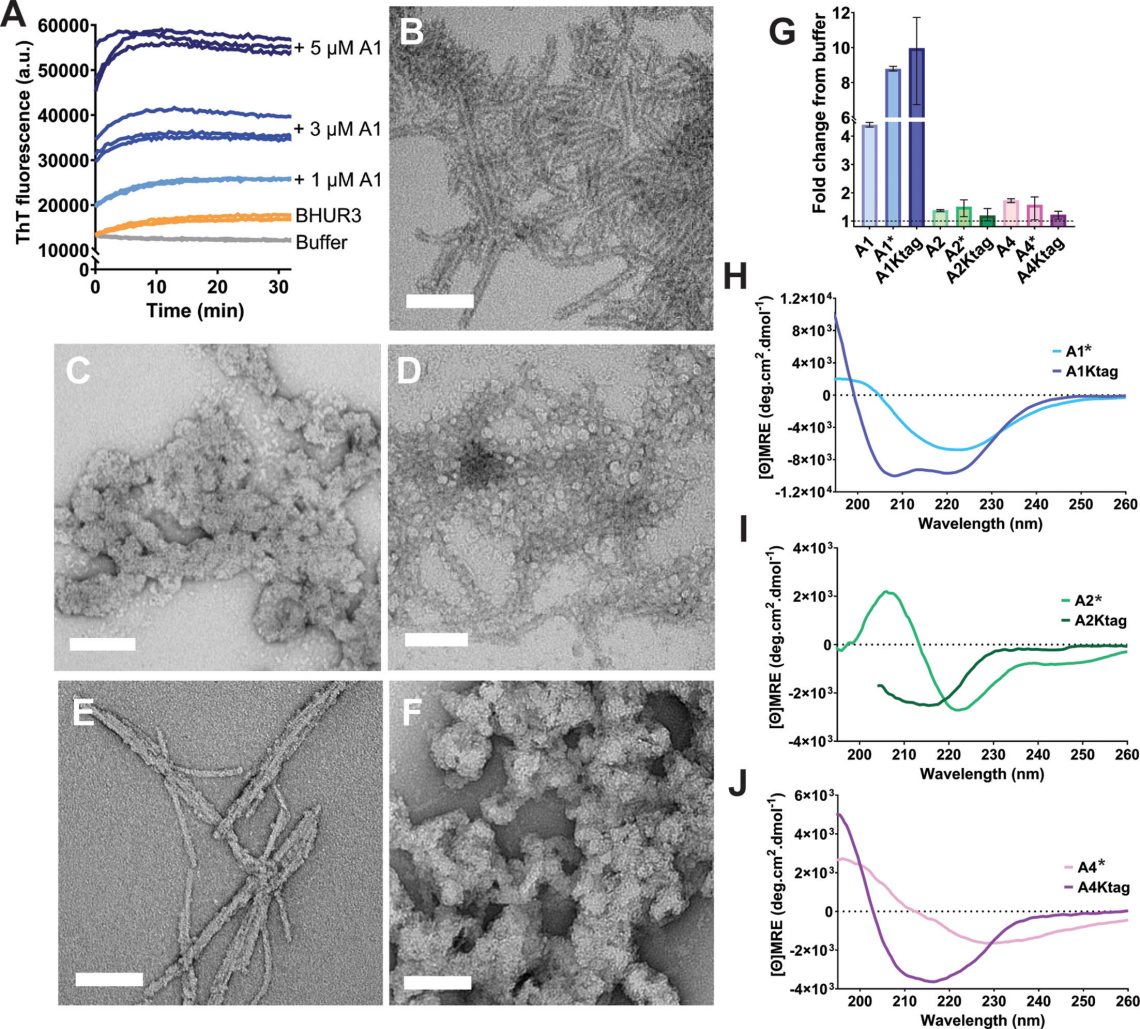
Influence of tetra-lysine tags on peptide aggregation propensity. (**A**) ThT traces of 1 μM BHUR3 assembling with 0–5 μM A1 peptide in TBS buffer. B–D) Representative TEM images showing B) 14 μM hRIPK3_387-518_ fibrils, (**C**) 2 μM A1 peptide, (**D**) 14 μM hRIPK3_387518_ fibrils incubated with 2 μM A1 peptide, (**E**) 14 μM insulin fibrils, and F) 14 μM insulin fibrils incubated with 2 μM A1 peptide. images taken at 110 000X magnification, scale bars represent 100 nm. Grids were stained using 2% uranyl acetate. (**G**) Average endpoint ThT signal after 1 h assembly in TBST showing the fold change for 2 μM peptide relative to buffer. Assay performed in triplicate, error bars represent range. H–J) CD spectra of tetra-lysine-tagged peptides compared to their matched K* constructs using 0.3 mg/ml H) A1*/A1Ktag, **I**) A2*/A2Ktag, and J) A4*/A4Ktag. Peptides were solubilised in HFIP then diluted into 10 mM Tris pH 8.0, 0.01% Tween 20, data show average of three collections. Data for A2Ktag were truncated due to interfering buffer components <205 nm.

The peptides were initially tested to determine whether they would influence amyloid assembly of hRIPK3, potentially acting as inhibitors if they preferentially bound to the monomeric form of hRIPK3 in a way that prevented RHIM:RHIM interactions. Unexpectedly, assay mixtures containing BHUR3 and any one of the peptides displayed a highly augmented ThT signal compared to BHUR3 alone (Supplementary Figure S2A and B), particularly peptide A1 (Figure 3A), though there was negligible change in the rate of increase of ThT fluorescence. Further examination revealed that samples of peptide alone generated a strong ThT fluorescence signal (Supplementary Figure S2C–E). However, the peptide-only oligomers lacked the fibrillar morphology of amyloid fibrils formed by BHUR3, instead forming irregular clusters ~ 50 nm in diameter (Figure 3B–C, Supplementary Figure S3A–B). When the peptides were incubated with preformed hRIPK3_387–518_ fibrils, the size of the peptide oligomers was markedly reduced. Peptide species of 5–10 nm were observed decorating the length of the fibrils, indicative of preferential interaction with hRIPK3 fibrils over self-assembly (Figure 3D, Supplementary Figure S3C–D). By contrast, when the peptides were incubated with insulin fibrils, they remained in large irregular clusters (Figure 3F, Supplementary Figure S3E–F).

The addition of lysine residues to introduce charge-charge repulsion between peptide monomers is a strategy that has been successfully employed by others to improve cyclic peptide solubility without compromising binding [21]. Therefore, each peptide was resynthesised with either a single C-terminal lysine residue to facilitate conjugation of a sulfo-Cy5 moiety (A1*, A2* and A4*), or with a tetra-lysine tag in place of the C-terminal linker sequence (A1Ktag, A2Ktag and A4Ktag). At the same time, the C-terminal cysteine in A1 and A4 was replaced by a serine to ensure correct cyclisation (see Table 1).

**Table 1:** Peptide sequences

Peptide	Peptide sequence
	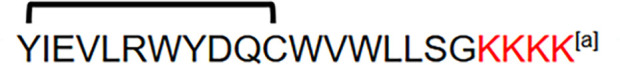
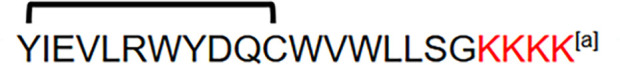	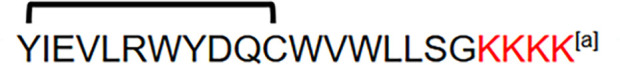
	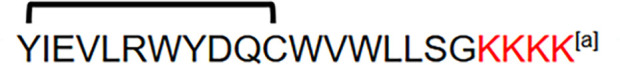
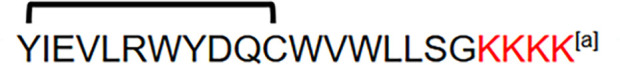	
	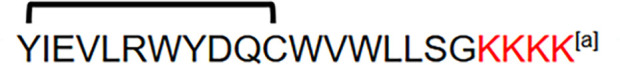
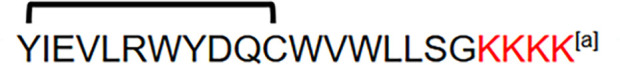	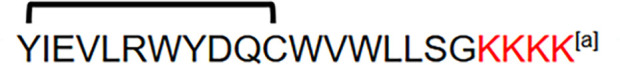
	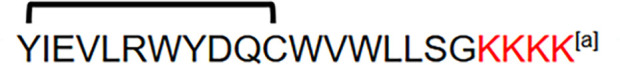
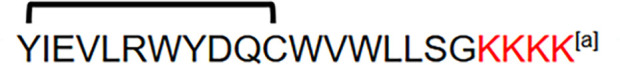	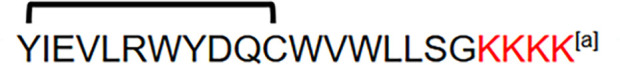
	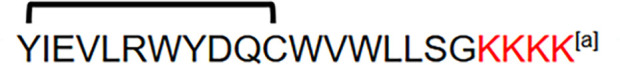

Amino acid sequences of original and modified peptides. Brackets denote cyclisation via thioether bond formation between *N*‑chloroacetyl‑L‑tyrosine and the closest cysteine residue. Modifications to original peptides shown in red. Peptides synthesised with and without a sulfo‑Cy5 label denoted with ^[a]^.

Single molecule photobleaching experiments demonstrated that with attachment of the sulfo-Cy5 label, the peptides were predominantly monomeric at 50 pM (Supplementary Figure S4). The introduction of the tetra-lysine sequence greatly reduced peptide self-assembly, as evidenced by reduction in ThT signal for A2Ktag and A4Ktag (Figure 3G), and by observation of smaller, more sparse assemblies by TEM (Supplementary Figure S5). Despite the increased solubility of A1Ktag compared to A1*, it still generated a strong ThT signal, possibly arising from an unusual interaction between this constrained cyclic peptide and the probe. This unusual interaction was further investigated using the alternate pan-amyloid-binding probe, Congo red and turbidity studies. For A1 and A2, no large shift in Congo red λ_max_ characteristic of amyloid formation was observed with or without tetra-lysine tags (Supplementary Figure S6), but turbidity was reduced. A4* showed some evidence of Congo red binding, as evidenced by higher absorbance at 540 nm than Congo red alone [25]. This was abrogated by the addition of the tetralysine tag, but turbidity was unaffected. These results suggest that tetra-lysine tags improve solubility of the peptides but do not affect the anomalous ThT binding.

Circular dichroism (CD) spectropolarimetry was used to investigate the conformations of the peptides in solution. The untagged peptides did not display a strong β-signal at 215 nm, as would be expected for conventional amyloid structures that display ThT binding. Instead, they show minima between 220 and 230 nm, with some aggregation observed during the collection of spectra (Figure 3H–J). All of the tetra-lysine-tagged peptides underwent a large conformation change and an increase in α-helicity resulting from the addition of the tetra-lysine tag. The observed increase in signal intensity was also indicative of reduced aggregation propensity. The tetra-lysine tagged variants, with or without the addition of a sulfo-Cy5 label, were therefore taken forward for all subsequent experiments.

### Identified cyclic peptides bind hRIPK3 with micromolar affinity

**Figure 4:**
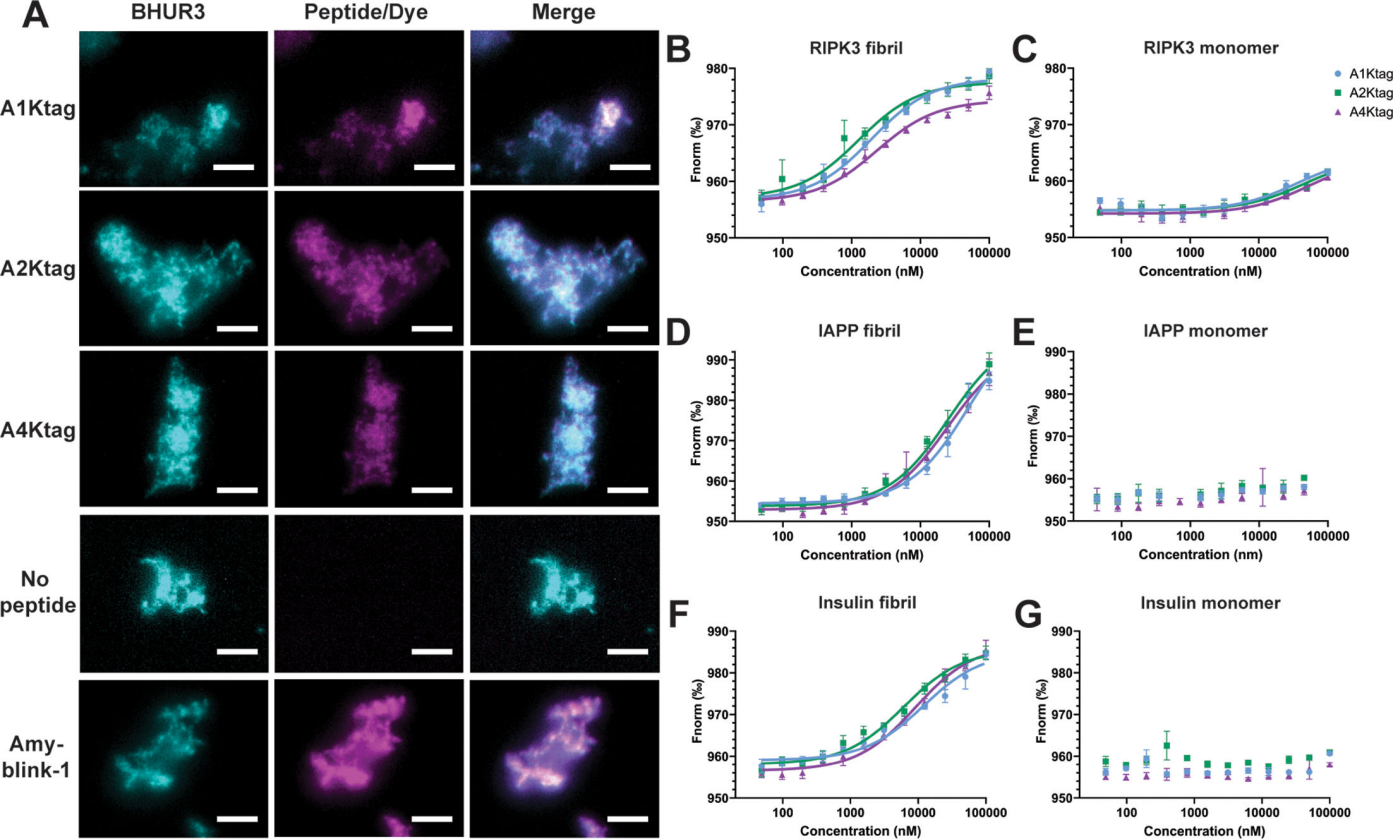
Binding of tetra-lysine-tagged peptides to hRIPK3_387–518_. (**A**) TIRF images of 500 pM sulfo-Cy5-lablled peptides with preformed BHUR3 fibrils. Fibrils were adhered to a poly-L-lysine-treated coverslip, then peptides, TBST or 50 nM Amyblink-1 diluted in TBST were incubated with fibrils for 15 min. Coverslips were washed thrice with TBST before imaging. Images taken at 100X magnification using 473 nm laser line (BHUR3) or 640 nm laser line (peptide/Amyblink-1), scale bars represents 5 μm. B–G) MST traces of 20 nM tetra-lysinetagged peptides with B) hRIPK3_387–518_ fibrils, (**C**) HUR3 monomer, (**D**) IAPP fibrils, (**E**) IAPP monomer, (**F**) insulin fibrils or G) insulin monomer. B and D–G were performed in TBST pH 8.0, 0.1 mg/ml BSA, 0.04% glycerol, 5% DMSO. C was performed in 4 M urea, 20 mM Tris pH 8.0, 5% DMSO to maintain HUR3 in a monomeric conformation. Assays performed in triplicate, *n* = 2 for all but G where *n* = 1. Points represent mean. Error bars indicate standard deviation.

TIRF fluorescence microscopy demonstrated binding of the sulfo-Cy5-labelled tetra-lysine-tagged peptides to hRIPK3_387-518_ fibrils immobilised on coverslips (Figure 4A). BHUR3 fibrils were imaged by autofluorescence arising from excitation at 473 nm. Autofluorescence was not observed with excitation at 640 nm. Amyblink-1 fluorescence reporter was used to validate the amyloid nature of the BHUR3 assemblies [26]. Subsequently, these peptides were used to determine affinities for hRIPK3_387-518_ fibrils using microscale thermophoresis (MST). All displayed low micromolar affinity, calculated on the starting hRIPK3_387–518_ monomer concentration, with dissociation constant (*Kd*) values of 1.96 ± 0.27, 1.26 ± 0.33 and 2.30 ± 0.39 μM for A1Ktag, A2Ktag and A4Ktag, respectively (Figure 4B). This analysis presumes a 1:1 binding mode; however, this stoichiometry is unlikely due to the self-assembling nature of both the peptides and their amyloid target. Given the relatively large size of the cyclic peptides, the binding site may span more than one hRIPK3 layer of the fibril.

To investigate selectivity, the peptides were exposed to preformed human islet amyloid polypeptide (IAPP) or insulin fibrils, and to monomeric forms of hRIPK3_387-518_, IAPP, insulin and hen egg white lysozyme. The hRIPK3_387518_ construct is extremely amyloidogenic and could only be maintained in a monomeric form at pH 8.0 in the presence of 4 M urea (Supplementary Figure S7A). Strikingly, some binding to monomeric hRIPK3_387–518_ was observed (Figure 4C), albeit weak ( > 35 μM), while no binding to monomeric IAPP, insulin or lysozyme was detected (Figure 4E and G and Supplementary Figure S7B), respectively. The affinity of the peptides for monomeric hRIPK3 under physiological conditions is likely to be higher than measured here, since hydrophobic interactions and H-bonding interactions are disfavoured in the presence of the denaturant. An interaction with IAPP fibrils (Figure 4D) and insulin fibrils (Figure 4F) was detected, though these did not achieve saturation at the concentrations used for hRIPK3_387–518_ fibrils, indicating an affinity for these fibrils of likely > 5 μM (all calculated *Kd* values available in (Supplementary Table S1). This is indicative of a general affinity for an amyloid structure.

### hRIPK3-binding peptides are non-toxic, cell-permeable and reveal the location of hRIPK3 in cells undergoing necroptosis

**Figure 5:**
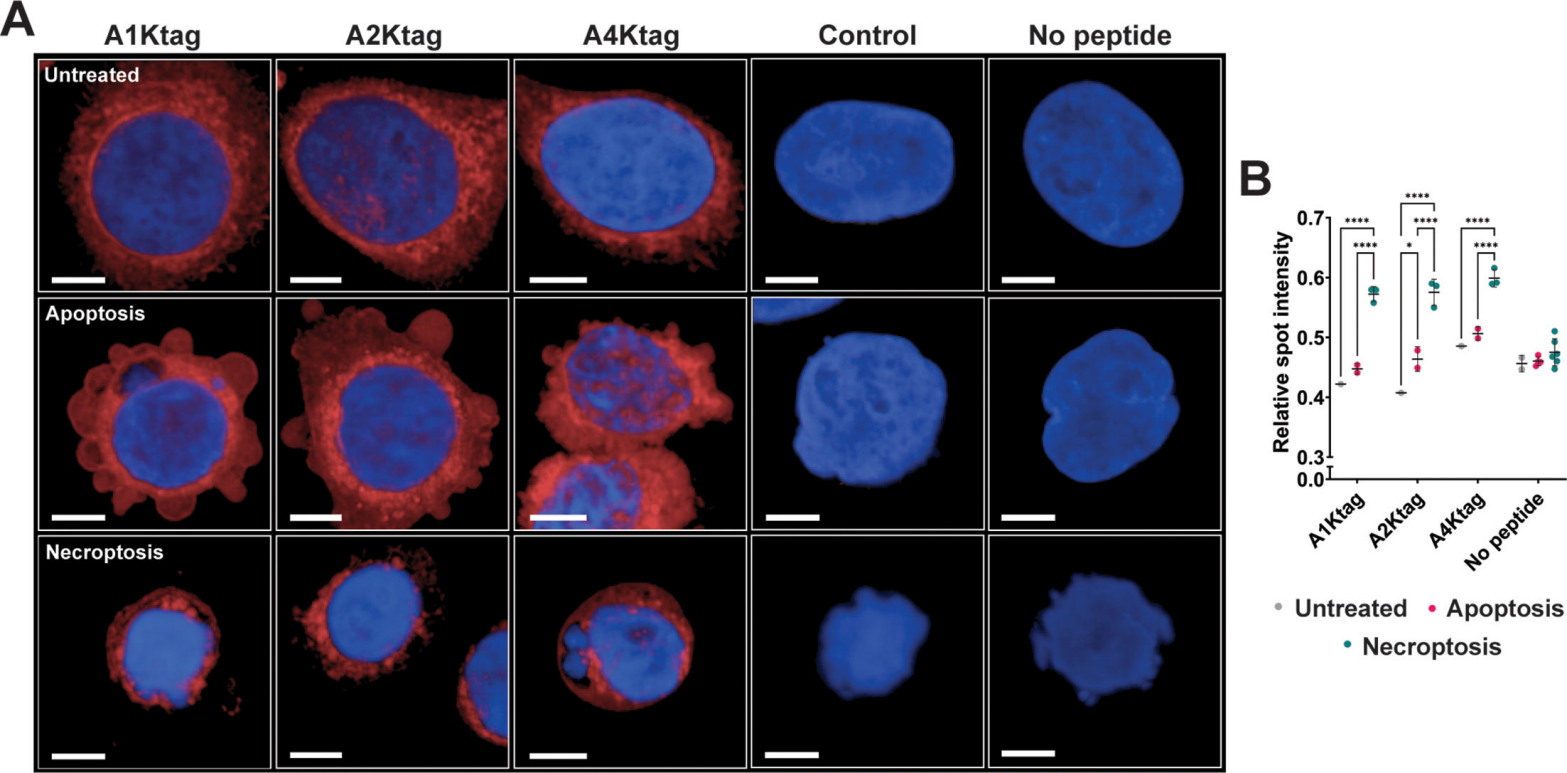
Sulfo-Cy5 labelled tetra-lysine-tagged peptides visualised in HT29 cells. **(A**) Confocal images of HT29 cells treated with DMSO vehicle (Untreated), TNFα and BV6 (Apoptosis) or TNFα, BV6 and zVAD-fmk (Necroptosis), fixed, permeabilised then incubated with 10 nM sulfo-Cy5labelled peptides (red) and DAPI (blue). Control peptide labelled with Alexa Fluor^TM^ 594 and imaged using appropriate excitation wavelenth. Images collected at 63X magnification, scale bar represents 10 μm. (**B**) Mean relative spot intensity within cells of each well as a ratio of spot intensity to background intensity. Mean and SD shown, data points represent each replicate well as a single biological replicate was performed. Number of cells analysed for each condition provided in (Supplementary Table S2). DMSO vehicle had single well, apoptosis duplicate wells and necroptosis triplicate wells for each peptide treatment. Two-way ANOVAs using Tukey’s multiple comparisons test conducted for statistical analysis (* < 0.05, ** < 0.01, *** < 0.001, **** < 0.0001). Non-significant comparisons not indicated.

HT-29 cells provide a well-established system to model necroptotic cell death, as they express the cellular machinery required for apoptosis and necroptosis [23,27,28]. This cell line was therefore selected to determine if the peptides could be used to visualise the localisation of hRIPK3 following induction of these cell death pathways. First, peptides were delivered after permeabilisation of the cell membrane. HT29 cells were treated with vehicle (DMSO, untreated control) or with TNF and the SMAC mimetic BV-6 to trigger apoptosis, or TNF, BV-6 and the pan-caspase inhibitor z-VAD to trigger necroptosis (TSZ). Following a 6-h incubation, cells were fixed, permeabilised and fluorescently labelled peptides were applied at 10 nM and then cells were extensively washed to remove unbound or weakly bound material. All three hRIPK3-binding peptides were able to enter the permeabilised cells (Figure 5A). The A1Ktag, A2Ktag and A4Ktag peptides were each observed inside all cells, regardless of the treatment condition. This contrasts with the lack of uptake or binding of a peptide identified by a RaPID screen against an unrelated protein (the transmembrane serine protease, TMPRSS2) not expected to be present in HT-29 cells, which was also tested and displayed no binding in these cells under any condition. Cells exposed to the control peptide labelled with an Alexa Fluor^TM^ 594 fluorophore were indistinguishable from cells not exposed to any sulfo-Cy5-labelled peptides.

The hRIPK3-binding peptides in untreated and apoptosis-induced cells were predominantly dispersed throughout the cytoplasm. Some perinuclear concentration was observed, consistent with studies of murine RIPK3 and imaging of hRIPK3-associated proteins [20,28]. The distribution of the peptides highlighted the characteristic blebbing morphology associated with cells undergoing apoptosis. In contrast, in the cells undergoing necroptosis, the peptides were concentrated into puncta which occupied most of the cytoplasm, consistent with the formation of hRIPK3-rich necrosomes. Analysis of the intensity of the Cy5 signal in observed puncta within cells demonstrated that the relative spot intensity in cells undergoing necroptosis was significantly higher than in untreated cells and in cells undergoing apoptosis (Figure 5B).

**Figure 6:**
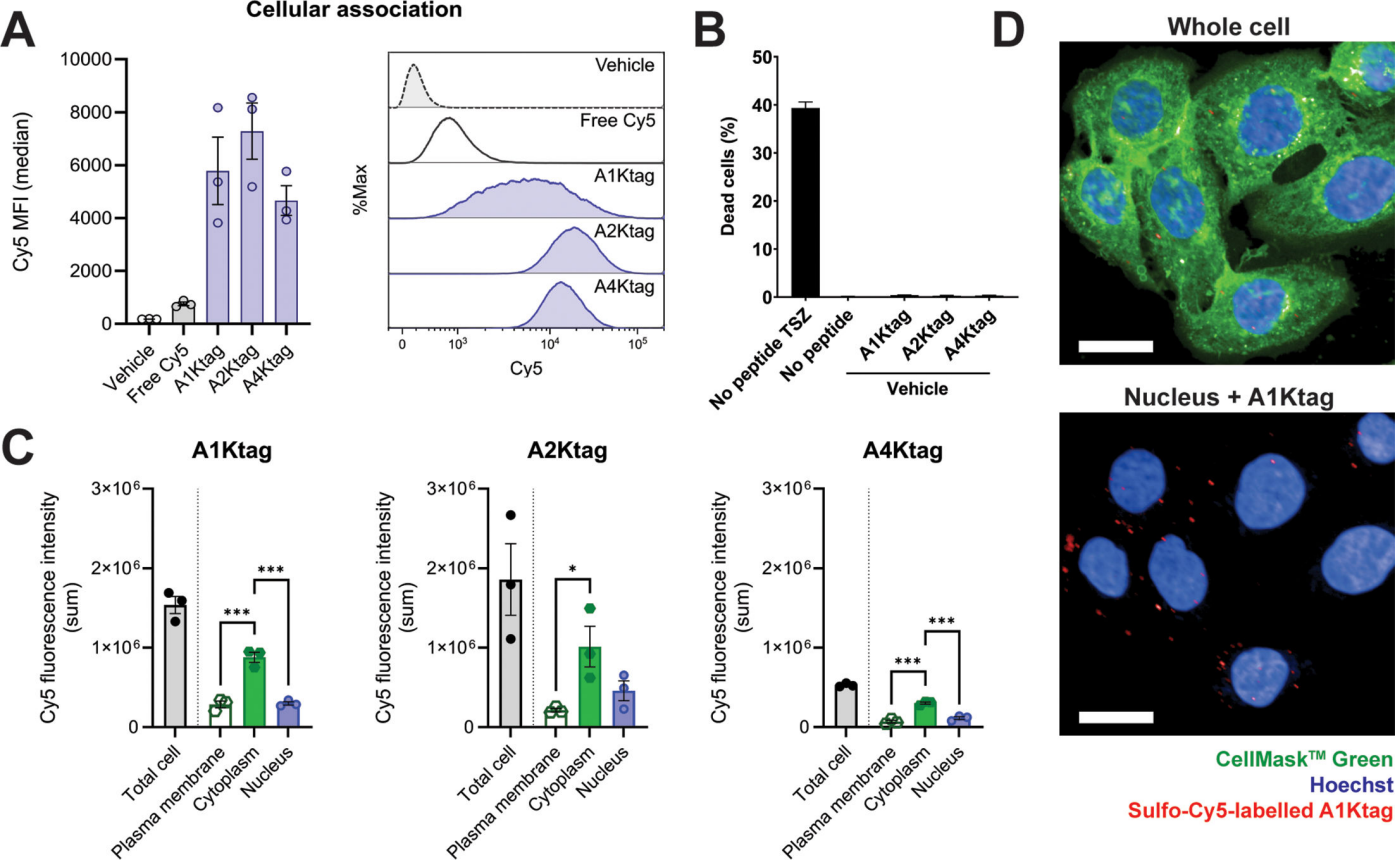
Incubation of sulfo-Cy5-labelled tetra-lysine-tagged peptides with live HT-29 cells. (**A**) Cellular association of 20 nM sulfo-Cy5-labelled peptides by flow cytometry showing median Cy5 intensity (left) and histogram of percent maximum Cy5 intensity (right). Data points represent average of each independent experiment, *n* = 3. (**B**) Mean percentage cell death detected by 2D imaging of propidium iodide staining following an 8-h incubation of 50 nM sulfo-Cy5 labelled peptides with live HT29 cells compared with cells treated for necroptosis. A single biological replicate was performed in triplicate. Error bars show SEM. (**C**) Analysis of peptide location within cells as the mean sum of Cy5 fluorescence intensity calculated from 3D imaging following a 4-h incubation with live HT-29 cells. Data points represent average of each independent experiment, *n* = 3. Error bars show SEM. One-way ANOVAs using Tukey’s multiple comparisons test conducted for statistical analysis (* < 0.05, ** < 0.01, *** < 0.001, **** < 0.0001). Non-significant comparisons not indicated. D) Maximum intensity projection of an example image used for analysis in (C) showing 20 nM Cy5-labelled A1Ktag peptide (red) and Hoechst nuclear stain (blue) with (top) and without (bottom) CellMask^TM^ Green plasma membrane stain (green). Images taken at 63X magnification, scale bar represents 20 μm.

We then sought to assess the cell permeability and uptake of tetra-lysine-tagged peptides when incubated with live HT29 cells. Peptide cell association with live cells was assessed via flow cytometry after a 4-h incubation of peptides with cells in media. Flow cytometry showed high levels of peptide association compared to free Cy5 (Figure 6A). We determined that the three hRIPK3-binding peptides are also not cytotoxic to live cells. HT-29 cells were incubated with the sulfo-Cy5-labelled peptides in media for 10 h, and no cell death was detected, as with no peptide (vehicle) controls (Figure 6B). These data contrasted with cells treated with TSZ to induce necroptosis, which showed ~40% cell death 8 h post-treatment. Along with the observed differences in peptide location and clustering between vehicle-treated, apoptosis and necroptosis conditions, these results are suggestive of hRIPK3 binding. Further experiments utilising hRIPK3 KO cells, or immunoprecipitation of the peptides from cell lysates treated to induce distinct cell death pathways, followed by mass spectrometry identification of bound proteins, will be required to determine the specificity of binding.

The lack of cytotoxicity encouraged us to investigate peptide uptake into live cells further. Confocal microscopy was used to determine whether the peptides were taken up into the cells and their subcellular distributions. All three hRIPK3-binding peptides showed cellular uptake, with the majority of each peptide located within the cytoplasm (Figure 6C) and Supplementary Figure S8. Some peptide was observed concentrated in organelles (e.g. endosomes), and a small amount was identified within the nucleus (**Figures 6C**) and Supplementary Figure S8. Future work is required to confirm the uptake pathways. In addition, if the peptides do not impact necrosome assembly, the hRIPK3-binding peptides could be used to track necrosome assembly and fate in live cells.

## Discussion

Self-assembling amyloidogenic proteins are challenging targets. Many are intrinsically disordered as monomers; hence, identification of molecules that bind to them through enthalpy-driven mechanisms is difficult. The end-point fibrils have a stable, ordered cross-β core but may be surrounded by flexible N- or C-termini. Along the course of fibril assembly, a heterogeneous mixture of oligomeric species is populated. In spite of these obstacles, cyclic peptides present promising amyloid-binding candidates because they have a constrained structure and will therefore experience a relatively small entropic loss upon binding to flexible regions, while offering the potential for many interactions with the extensive fibril surface. Mirror-image phage display and phage-assisted continuous evolution (PACE) have previously identified cyclic peptides that bind and inhibit formation of disease-associated amyloids [29–31].

Here, we have successfully applied RaPID mRNA display to identify the first cyclic peptide binders for a ***functional amyloid***, hRIPK3, during the process of amyloid formation. During the selection, multiple species of hRIPK3 are expected to be populated, namely monomer, small oligomers and mature fibrils. By MST, the identified peptides were shown to bind to both monomeric and fibrillar forms of purified hRIPK3_387-518_. Likewise, in the cell-based experiments, binding of peptides to hRIPK3 was observed under conditions where the protein is monomeric (DMSO-only and apoptosis treatment) and when hRIPK3 is in the amyloid fibril form (necroptosis treatment).

**Figure 7:**
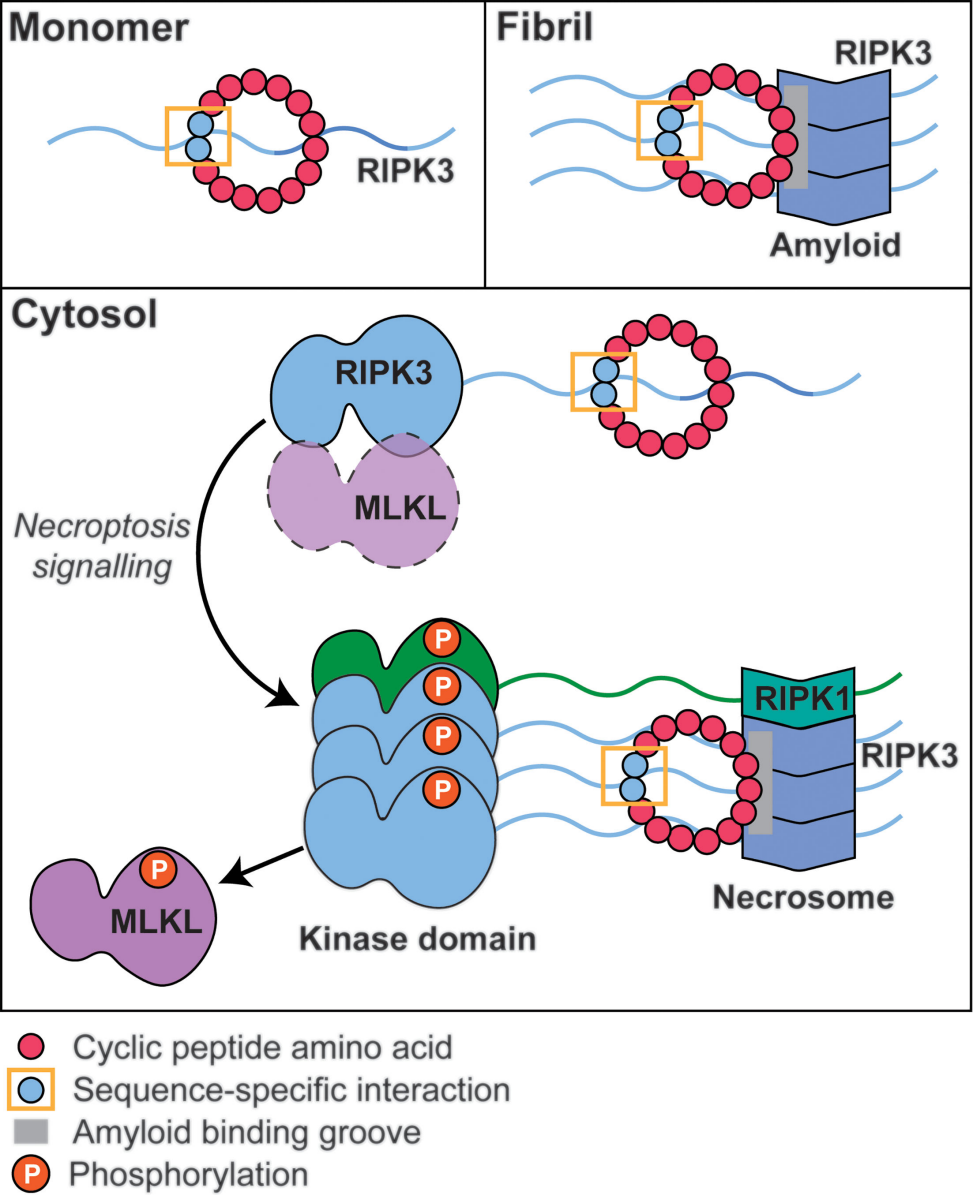
Model for binding of peptides to RIPK3. Cyclic peptides (red) can bind to RIPK3 via sequence-specific interaction (boxed in yellow), and non-specifically within grooves of the amyloid fibril fold (grey). Within the cytosol, peptides can bind to the monomeric form of RIPK3 (in complex with the pseudokinase MLKL) and to the heteromeric amyloid necrosome structure in response to necroptotic stimulus. Multiple copies of the cyclic peptides would be expected to bind along the length of the amyloid fibril.

These results indicate that the site of sequence-specific interaction of the peptides with hRIPK3 lies in a region which is accessible and has a similar conformation in both monomeric and fibrillar forms (Figure 7). The structure of the cross-β core of hRIPK3 fibrils is known from ssNMR and cryoEM studies [16]. During fibril assembly, the ~18-residue RHIM of hRIPK3 adopts a stable three β-stranded 2D fold, with one copy of hRIPK3 occupying each layer of the fibril, separated by 4.7 Å. This arrangement brings the regions adjacent to the RHIM into close proximity. There is solution NMR evidence for a relatively structured ‘pre-RHIM’ region of ~20 residues immediately N-terminal of the RHIM [17]. Further experiments will be necessary to determine whether the unique RIPK3 site bound by the peptides is related to the pre-RHIM region.

The peptides also showed some affinity for IAPP and insulin fibrils. As a consequence of the common cross-β structure that is a defining characteristic of amyloid [32], all fibrils have similar grooves on the surface of the fibril core. Pan-amyloid reporter molecules such as Congo red and Thioflavin T bind into these grooves. The observed interactions of these peptides with multiple fibril types are consistent with the presence of a generic secondary peptide binding site. In hRIPK3, this may augment the binding from the specific site (Figure 7). In mammalian cells, there are three other proteins that contain RHIMs, RIPK1, TRIF and ZBP1. The ~20 residues of the RHIM adopt similar amyloid structures to RIPK3, hence are likely to show some generic interaction with these peptides in the assembled, fibrillar state like IAPP and insulin. These proteins only adopt the amyloid form following induction of necroptosis. The regions in these proteins that flank the RHIMs are not homologous to the RIPK3 sequence, so sequence-specific interactions are not expected. However, definitive investigation of the specificity of the peptides will require identification of peptide-binding proteins from cell lysates.

MST analysis showed binding of peptides to the monomeric form of hRIPK3 but not to the monomeric form of any other tested amyloid-forming protein. Binding to hRIPK3 occurred even in 4 M urea, which was required to prevent fibril formation of this highly amyloidogenic protein. This suggests a very strong association with a specific site within the hRIPK3_387-518_ sequence. While it was not possible to determine an accurate *Kd* for the monomeric form of hRIPK3 under native-like conditions due to the amyloidogenicity of the RHIM, strong binding was observed even with the use of nanomolar concentrations of the peptides in cell-based studies. This suggests that the affinity for hRIPK3 may be stronger than measured here. The RaPID approach commonly identifies cyclic peptide binders with nanomolar affinity for their target [33–35].

In another study, peptides were identified from a RaPID screen against mature αsynuclein fibrils [36]. Those peptides exhibited dissociation constants of ~3–10 μM and displayed the ability to induce liquid–liquid phase separation in αsynuclein, and under some conditions accelerated amyloid fibril formation [36]. Similar to the hRIPK3-binding peptides, not all identified peptides were specific for α-synuclein, highlighting that self-assembling proteins share similar structural features or properties, from the sticker residues of phase separating proteins to the grooves present on cross-β amyloid fibrils [37].

The accessibility of the RIPK3 binding site outside of the amyloid core is supported by observations in cell-based experiments. Peptide binding to hRIPK3 was observed under all trialled conditions (vehicletreated, apoptosisinduced and necroptosisinduced), even though the distribution and pattern of peptide localisation was distinct across each condition, indicating strong peptide binding to full length hRIPK3 both free within the cytosol, and assembled into a heteromeric amyloid necrosome (Figure 7). Previous studies have examined the suitability of antibodies and monobodies as probes for hRIPK3; however, no antibodies suitable for immunofluorescence could be identified [28]. Understanding of the activity and interactions of this important human kinase have been limited by the lack of effective reporters for use in live cells. The non-cytotoxic and cell-permeable nature of these peptide binders allows them to be used to monitor hRIPK3 in cells under normal physiological conditions and to track hRIPK3 during programmed cell death. These peptides therefore represent a useful starting point for the development of tools that can report on localisation and intracellular partners of hRIPK3 and be adapted to modulate its activity for therapeutic outcomes.

## Methods

### Production of biotinylated (BHUR3) and non-biotinylated (HUR3) RHIM domain of hRIPK3

The RHIM-containing domain of hRIPK3 (hRIPK3_387-518_) was expressed as a His_6_-ubiquitin-TEV-RIPK3_387-518_ construct (HUR3) or biotinylated His_6_-ubiquitin-TEV-RIPK3_387-518_ (BHUR3) in BL21(DE3) pLysS *E. coli* (Novagen), with cells grown at 37 °C for 3 h after induction with 0.5 mM IPTG at mid-log phase. For complete biotinylation, 100 μM biotin was added during IPTG induction of BHUR3. BHUR3 and HUR3 were purified by Ni-NTA affinity chromatography under denaturing conditions. Expression pellets were resuspended in 20 mM Tris pH 8.0, 150 mM NaCl, 1 mM EDTA and lysed by sonication. Lysate was centrifuged at 26000*×*g for 45 min at 4 °C and insoluble material retained and resuspended in 6 M GuHCl, 20 mM Tris pH 8.0 with harsh agitation at room temperature (RT) until fully dissolved. Guanidine solution was centrifuged again at 26000*×*g for 30 min at 4 °C. Then, 1 mM β-mercaptoethanol was added and supernatant taken forward for Ni-NTA affinity chromatography. B(HUR3) was buffer exchanged into 8 M urea, 20 mM Tris, 100 mM NaH_2_PO_4_ occurred on the column and BHUR was eluted from the affinity resin by reduction of buffer to pH 4.0. Protein was concentrated to >200 μM using 10 kDa MWCO spin filters (Millipore), then buffer exchanged into 8 M urea 20 mM sodium acetate pH 4.0 and stored at 4 °C to avoid unwanted self-assembly. Concentration of BHUR3 and HUR3 was determined by A_280_ measurement (using extinction coefficients of 34 950 M^–1^.cm^–1^ for BHUR3, 29 450 M^–1^.cm^–1^ for HUR3. Reagents were purchased from Sigma Aldrich unless otherwise indicated.

### BHUR3 fibril formation and removal of His_6_-ubiquitin

(Biotin)-His_6_-ubiquitin-TEV-RIPK3_387-518_ was diluted into 8 M urea, 20 mM sodium acetate pH 4.0 [1] to ≤100 μM protein concentration and dialysed against 25 mM NaH_2_PO_4_, 150 mM NaCl (SnakeSkin dialysis tubing 3.5 kDa MWCO) containing 0.5 mM DTT for 1 h at RT, then Tobacco Etch Virus (TEV) enzyme (produced in house) was added at minimum of 75 μg/ml final concentration and samples were dialysed overnight with gentle magnetic stirring. Samples from before and after dialysis were analysed by SDS PAGE to assess extent of TEV cleavage. If incomplete, the sample was resolubilised and this process was repeated. Fibrils were recovered by centrifugation at 17000*×*g for 10 min and washed into desired buffer twice to remove TEV and (Biotin)His_6_ubiquitin tag, then stored for use at 4 °C. Cleaved BHU product was retained and concentration estimated for use in RaPID counterselection using *ε* = 8480 M^1^.cm^-1^.

### RaPID screening

Optimal bead loading concentrations were determined by incubating 2 pmol BHUR3 protein with 8, 4, 2, 1, 0.5 and 0.25 μl Streptavidin M280 DynaBeads^TM^ and analysing immobilisation efficiency by SDSPAGE with SYPRO™ Ruby protein stain. Optimal loading was determined by identifying the smallest volume of bead where no protein was observed in the supernatant fraction, indicating that all available protein had been immobilised.

For RaPID screening, a randomised cyclic peptide library was provided by Sydney Analytical Facility (The University of Sydney). This library was prepared using *in vitro* transcription and translation from DNA oligos containing a fixed T7 promoter, a ribosome binding site and a start codon (AUG) then a random series of 4–15 NNK codons (where *N* = A, T, C or G and K = C or G), followed by a fixed cysteine-encoding codon (TGC) and region encoding a GNLI linker sequence. Briefly, *in vitro* transcription using T7 RNA polymerase was performed, and the resulting mRNA library was purified by denaturing urea polyacrylamide gel electrophoresis. mRNA library was ligated to a puromycin-linked oligonucleotide via T4 RNA ligase. mRNA-puromycin library (1 μM) was then translated to generate the cyclic peptide library via ribosomal synthesis using the PURExpress ΔRF kit (NEB), lacking RF1 in a Met-deficient system. This library was prepared using 25 μM initiating tRNA that had undergone aminoacylation with N-chloroacetyl L-tyrosine using eFx flexizyme such that all starting residues were translated as N-chloroacetyl L-tyrosine to allow for thioester formation with downstream cysteine residues [24,31]. After 30 min at 37 °C, ribosomes were denatured by addition of 20 mM EDTA and peptides released into solution over another 30 min at 37 °C. mRNA-linked cyclic peptides subsequently underwent reverse transcription with M-MLV RNase (H–) reverse transcriptase (Promega) using appropriate reverse primer, then TBST selection buffer (20 mM Tris. HCl pH 8.0, 150 mM NaCl) containing 0.5 mM DTT was added so that final library volume was 300 μl. A small sample of this library (input) was reserved and diluted 100-fold for RTqPCR analysis.

For round 1 of RaPID screening, BHUR3 was diluted out of denaturant into starting cyclic peptide library to a final concentration of 0.8 μM. This mixture was gently rotated at RT for 20 min, then overlayed onto Streptavidin M280 DynaBeads^TM^ that had been prewashed thrice with selection buffer (0.25 μl bead slurry/pmol protein). This mixture was rotated at RT for a further 5–10 min to pull down biotinylated protein and bound peptides, then supernatant was removed. Beads were washed thrice in an equal volume of selection buffer, changing tubes each wash, then resuspended in 100 μl 0.1% Triton X-100 and boiled at 95 °C for 5 min to recover bound peptides. A small sample (1 μl) peptide recovery mixture was reserved for RT-qPCR analysis.

For all rounds after the first, counterselection was performed prior to positive selection using TEV-cleaved BHU protein. 6X bead volume required for screening was washed thrice with selection buffer, then incubated with 0.8 μM BHU for 30 min at RT gently rotating, then washed twice with selection buffer again. Beads were split into three aliquots (50 μl), and each was incubated with the prepared cyclic peptide library for 30 min gently rotating at RT to remove non-specific binders. Final bead aliquots were washed and recovered for RT-qPCR analysis as previously described. Positive selection for all subsequent rounds of screening occurred as for round 1 but in 50 μl total volume.

Recovered DNA was amplified by PCR with appropriate primers, and once sufficient DNA product was visualised, the PCR reaction products were precipitated out with 0.1 vol 3 M NaCl and 2.2 vol 100% ethanol. Precipitated DNA was recovered by centrifugation at 12000×g for 10 min, then washed with 70% (v/v) ethanol. Pellet was allowed to air dry, then resuspended in MilliQ water (10 μl). Resuspended DNA was used for transcription, with reactions containing reserved DNA (5 μl), 1X T7 buffer, DTT (10 mM), MgCl_2_ (30 mM), (5 mM ea.) and 1X T7 RNA polymerase. Reaction mixtures were prepared on ice and then incubated at 37 °C overnight. The reaction was quenched by the addition of NaCl (300 mM) and EDTA (25 mM), then mRNA was precipitated by addition of ⅞ volume 2-isopropanol and recovered by centrifugation at 15000×g for 5 min. Pellet was washed with 70% ethanol and then allowed to air dry. mRNA pellet was resuspended in MilliQ water (20 μl), and concentration was determined by A_260_ signal (average mRNA length 106 nt, molecular weight 340 Da). mRNA was diluted to 10 μM in MilliQ water and puromycin was ligated to transcribed mRNA. Puromycin ligation reactions contained 1X T4 buffer, prepared mRNA library (1 μM) and 0.15 U/μl T4 RNA ligase. Ligations occurred during incubation at 37 °C for 30 min then were quenched and precipitated by the addition of 300 mM NaCl, 25 mM EDTA and 250 μg/ml glycogen followed by 4 vol of 2-isopropanol. Precipitated mRNA puromycin conjugates were pelleted by 15 min centrifugation at 15000×g, washed once with 70% ethanol, then air dried. The pellet was resuspended in 2 μl MilliQ water to yield a stock concentration of ~6 μM. Translation of mRNA-puromycin libraries for all subsequent rounds was performed as for round 1 but at a 1/20 scale. Following translation, reverse transcription was completed by adding 250 μM ea. dNTPs, 15 μM Mg(OAc)_2_, 25 mM Tris pH 8.0, 10 mM KOH, 2 μM reverse primer and 1X MMLV RT (H–). Reaction was allowed to incubate at 37 °C for 30 min, then the cyclic peptide library was diluted to 50 μl in TBST buffer containing 0.5 mM DTT. Positive recovery samples for all rounds were prepared and sent for sequencing by iSeq at the Ramaciotti Centre for Genomics at the University of New South Wales.

### General materials and methods for peptide synthesis

Peptide grade *N,N*-dimethylformamide (DMF) for peptide synthesis was purchased from RCI. Gradient grade acetonitrile (MeCN) for chromatography was purchased from Sigma Aldrich and ultrapure water (Type 1) was obtained from a Merck Millipore Direct-Q 5 water purification system. Standard fluorenylmethoxycarbonyl (Fmoc)-protected amino acids (Fmoc-Xaa-OH), coupling reagents and resins were purchased from Mimotopes or Novabiochem. Fmoc-PEG_2-_OH and Fmoc-Cha-OH were purchased from GL Biochem (Shanghai) Ltd and AK Scientific, respectively. Piperidine and *N,N*-di*iso*propylethylamine (DIPEA) were purchased from Alfa Aesar and Merck, respectively. Fmoc-SPPS was performed through automated synthesis on a Syro I peptide synthesizer (Biotage). Sulfo-Cy5 DBCO was purchased from BroadPharm. All other reagents were purchased from Sigma Aldrich, AK Scientific or Merck and used as received.

### Preparative liquid chromatography

Preparative and semi-preparative reversed-phase high performance liquid chromatography (RP-HPLC) was performed using a Waters 600E multisolvent delivery system with a Rheodyne 7725i injection valve (5 ml loading loop) with a Waters 500 pump and a Waters 490E programmable wavelength detector operating at 214 nm and 280 nm. Preparative reversed-phase HPLC was performed using a Waters X-Bridge® C18 OBD^TM^ Prep Column (5 µm, 30 × 150 mm) at a flow rate of 38 ml min^-1^ using a mobile phase of 0.1% TFA in water (solvent A) and 0.1% TFA in MeCN (solvent B) on linear gradients, unless otherwise specified.

### Liquid chromatography-mass spectrometry

Liquid Chromatography-Mass Spectrometry (LC-MS) was performed on a Shimadzu 2020 UPLC-MS instrument with a Nexera X2 LC-30AD pump, Nexera X2 SPD-M30A UV/Vis diode array detector and a Shimadzu 2020 (ESI) mass spectrometer operating in positive ion mode. Separations were performed on a Waters Acquity BEH300 1.7 µm, 2.1 × 50 mm (C18) column at a flow rate of 0.6 ml min^-1^. All separations were performed using a mobile phase of 0.1 vol.% formic acid in water (solvent A) and 0.1 vol.% formic acid in MeCN (solvent B) using linear gradients over 5 min.

### Analytical RP-HPLC

Analytical reversed-phase HPLC was performed on a Waters Alliance e2695 HPLC system equipped with a 2998 PDA detector (λ = 210–400 nm). Separations were performed on a Waters XBridge® Peptide BEH300 5 µm, 4.6 × 250 mm (C18) column at 40 °C with a flow rate of 1.0 ml min^-1^. All separations were performed using a mobile phase of 0.1% TFA in water (Solvent A) and 0.1% TFA in MeCN (Solvent B) using linear gradients, unless otherwise specified. Analytical RP-HPLC traces were processed where time 0 min refers to the start of the gradient.

### Mass spectrometry

Low-resolution mass spectra were recorded on a Shimadzu 2020 (ESI) mass spectrometer operating in positive and negative mode. High-resolution mass spectra were recorded on a Bruker-Daltronics Apex Ultra 7.0 T Fourier transform (FTICR) mass spectrometer.

### Peptide synthesis

**General procedure A;** Automated Peptide Synthesis (SYRO I peptide synthesizer): The resin (90 mg, 50 µmol, 0.56 mmol g^-1^, 1 eq.) was treated with 40 vol.% piperidine in DMF (800 µl) for 4 min, drained, then treated with 20 vol.% piperidine in DMF (800 µl) for 4 min, drained and washed with DMF (4 × 1.2 ml). The resin was then treated with a solution of Fmoc-Xaa-OH or chloroacetic acid (200 µmol, 4 eq.) and Oxyma (220 µmol, 4.4 eq.) in DMF (400 µl), a 1 wt.% solution of 1,3-diisopropyl-2-thiourea in DMF (400 µl), followed by a solution of DIC (200 µmol, 4 eq.) in DMF (400 µl). Coupling of Fmoc-Cys(Trt)-OH and Fmoc-His(Trt)-OH was carried out at 50°C for 30 min. All other coupling reactions were conducted at 75°C for 15 min. The resin was then drained and washed with DMF (4 × 1.2 ml) before being treated with a solution of 5 vol.% Ac_2_O and 10 vol.% *i*Pr_2_NEt in DMF (800 µl) for 6 min at room temperature, drained and washed with DMF (4 × 1.2 ml).

**General procedure B**: Manual cleavage: The resin was thoroughly washed with CH_2_Cl_2_ (5 × 5 ml) before being treated with 87.5:5:5:2.5 v/v/v TFA:tri*iso*propylsilane:H_2_O:EDT (5 ml) and shaken at room temperature for 2 h. The resin was filtered, and the filtrate concentrated under a stream of nitrogen before addition of diethyl ether (40 ml). The peptide was pelleted by centrifugation (4 min, 4 °C, at 5000*×*g) and the ether was decanted. The crude peptide was dissolved in the minimum volume of 1:1 MeCN/H_2_O and concentrated by lyophilisation.

**General procedure C:** Manual cleavage: The resin was thoroughly washed with CH_2_Cl_2_ (5 × 5 ml) before being treated with 90:5:5 v/v/v TFA:tri*iso*propylsilane:H_2_O (5 ml) and shaken at room temperature for 2 h. The resin was filtered, and the filtrate concentrated under a stream of nitrogen before addition of diethyl ether (40 ml). The peptide was pelleted by centrifugation (4 min, 4 °C, at 5000*×*g) and the ether was decanted. The crude peptide was dissolved in the minimum volume of 1:1 MeCN/H_2_O and concentrated by lyophilisation.

**General procedure D:** Cyclisation: The crude peptide (25 µmol) was dissolved in DMSO (5 ml) and *i*Pr_2_NEt (180 µl) was added. The peptide solution was heated in a water bath at 60 °C until the cyclisation was complete as judged by UPLC-MS analysis.

**General procedure E;** Acm removal: The purified peptide (1.2 µmol) was dissolved in a 1:1 v/v mixture of MeCN and H_2_O with 0.1 vol.% TFA (0.1 M) and AgOAc (40 eq., 48 µmol) was added. The mixture was shaken at room temperature until the reaction was complete as judged by UPLC-MS analysis. Dithiothreitol (60 eq., 72 µmol) was added to the reaction prior to RP-HPLC.

**General procedure F:** Strain promoted alkyne-azide cycloaddition (SPAAC) reaction: The peptide was added to one molar equivalent of DBCO-fluorophore in a 1:1 v/v mixture of DMSO and H_2_O (approximately 10 mg/ml). The mixture was stirred at room temperature until the reaction was complete as judged by UPLC-MS analysis.

### Thioflavin T and turbidity assays

Solutions with appropriate buffering agent at pH 4.0–8.0 as specified, NaCl as required and containing 40 μM ThT were prepared in Corning^TM^ black clear bottom 96-well plates (cat no. 3631). Peptides and proteins were diluted from concentrated stocks into buffer immediately prior to measurement. Final urea concentration and DMSO volume did not exceed 200 mM and 1% unless otherwise specified. Plates were sealed with optically clear sealing film (Corning^TM^, cat no. 6575) and loaded into a BMG POLARstar Omega microplate reader maintained at RT or 37 °C. Plates were shaken for 30 s at 700 rpm before first read (unless otherwise specified), and wells were excited at 440 nm, with fluorescence emission measured at 480 nm (filters +/– 10 nm). The gain was set at 1–5% from a buffer and ThT-containing sample. Assays were continued until fluorescence emission plateaued. Turbidity assays were performed concurrently to ThT assays, with absorbance at 350 nm measured after ThT emission measurement.

### Negative stain transmission electron microscopy

Carbon-coated copper 200-mesh grids coated with formvar support film (ProSciTech Pty Ltd.) were glow discharged at 25 mA for 30 s at desired charge. Grids were floated on 20 μl droplets containing 10 μM peptide or 0.2 mg/ml hRIPK3_387–518_ for 2 min, washed briefly with filtered MilliQ water three times and then blotted and stained by floating on 2% aqueous uranyl acetate stain for 30–60 s and blotted dry. For pull-down experiments, fibrils were incubated with grids and washed as described, then grids were further floated onto droplets containing 2 μM peptide, washed, then stained as described. Transmission electron microscopy was performed using FEI Tecnai T12 electron microscope operating at 120 kV at the Sydney Microscopy and Microanalysis Core Research Facility. Images were captured using a Veleta CCD camera and RADIUS 2.0 imaging software (EMSIS GmbH) or a CMOS camera with the DigitalMicrograph Software (GATAN).

### Congo red assays

0.15 mg/ml peptide, insulin monomer or insulin fibril was diluted into TBS buffer with and without 1 µM Congo red to a final volume of 250 µl in a Grenier Bio-One clear flat-bottom 96-well plate (cat no. 655101). Absorbance was measured using a BMG VantaStar platereader. Plates were shaken for 10 s at 700 rpm to mix samples and absorbance was measured between 400–600 nm. Respective measurements for samples without Congo red were subtracted from samples containing Congo red, and resulting data were normalised to analyse spectral shifts.

### Circular dichroism spectroscopy

Experiments were performed using a Jasco J 815 CD Spectropolarimeter and Spectra Manager^TM^ software (Jasco). Samples contained a final concentration of 0.3 mg/ml peptide or matched buffer in a 1 mm High Precision Cell quartz cuvette (Hellma Analytics). Spectra were acquired with 0.5 nm step resolution from 260 to 190 nm, at 20 nm/min, with sample maintained at 25 °C. Spectra were collected using only one accumulation but were acquired three times for each sample, apart from buffer alone samples, which were acquired only once. Data were analysed using Spectra AnalysisTM software (Jasco). Buffer-alone spectra were subtracted from peptide or protein spectra and data smoothed using the Savitzky-Golay method. The secondary structure content was analysed using the BestSel online predictive software.

### Insulin fibril formation

Purchased, lyophilised human insulin (Sigma Aldrich, cat no. 91077C) was dissolved at 2.5–3 mg/ml in 20 mM glycine pH 2.0. This was stored at 4 °C and used as monomeric control or heated at 60 °C for 8 h with shaking at 800 rpm to convert to amyloid fibrils. Amyloid formation was confirmed by ThT assay.

### IAPP fibril formation

Purchased, lyophilised human IAPP (Abcam, ab142398) was dissolved in deionised water and concentration was calculated using A280 signal and the extinction coefficient (ε) 1615 M^-1^cm^-1^, then diluted into 10 mM Tris pH 7.5 at 70 μM and allowed to form quiescently at RT over 16 h. Amyloid formation was confirmed by ThT assay and TEM.

### Microscale thermophoresis

HUR3 was cleaved by TEV enzyme and assembled into fibrils as described previously. The sample was then buffer exchanged into TBST by two rounds of centrifugation at 17000×g for10 min, resuspending the fibril pellet in fresh TBST following each spin. Insulin monomer was prepared, and fibrils were formed as described previously. Insulin fibrils and monomeric protein were then diluted to 200 μM and dialysed overnight into TBS to avoid buffer mismatch. Lyophilized lysozyme (Sigma Aldrich) was dissolved in TBS, filtered with a 0.22 μm filter and diluted to appropriate starting concentration. IAPP fibrils and monomers were prepared as described previously. Fibrils were recovered and washed into TBST buffer via centrifugation at 21000×g for 30 min and concentrated to 200 μM for MST assays. IAPP monomer samples were doped with concentrated buffer solutions to achieve buffer matching and assays were performed immediately after thawing. RIPK3 and insulin fibril samples were probe sonicated immediately prior to serial dilutions with 2 × 20 s at 25% power, with 45 s rest on ice in between each sonication. IAPP fibrils were also sonicated immediately prior to serial dilutions at 15% power for 30 s total. Protein samples were serially diluted 1:1 into PCR tubes to make dilutions of 100–0.048 μM or 45–0.044 μM (IAPP monomer) in TBST containing 0.04% glycerol, 0.1 mg/ml BSA and sulfo-Cy5 labelled peptide so that total volume was 5 μl and final peptide concentration was 20 nM. Samples were incubated for 5–10 min then loaded into premium capillaries (NanoTemper) for measurement.

Microscale thermophoresis (MST) was conducted with a NanoTemper Monolith. For MST measurements, excitation power (red LED laser) was set to 95%, and MST power (infrared laser) was set to 20% and lasted 15 s. Fluorescence readings were taken for an additional 5 s before and after switching on the infrared laser. Each capillary was measured in triplicate, and the experiment was repeated once. Data were analysed using MO Affinity Analysis v2.3 utilising a *Kd* fit model (NanoTemper).

### TIRF fluorescence imaging

Cleaned glass coverslips (24 × 24 mm, 130–160 µm thickness, Epredia) were treated with poly-L-lysine solution (0.01%) immediately prior to use. RIPK3_387-518_ fibrils ( ≤ 5 µM) were incubated with poly-L-lysine-treated coverslips for 15 min then coverslips were washed thrice with TBST and blotted to remove unbound material. Sulfo-Cy5-labelled peptides (50–500 pM) or Amyblink-1 (50 nM) were diluted into TBST and incubated with coverslips in the dark for 20 min, then coverslips were washed thrice with TBST and blotted, then overlayed with 20 µl 50 mM Tris pH 8.0, 10 mM NaCl before mounting onto slides and sealing with epoxy resin for imaging.

TIRF imaging was performed using an ONI Nanoimager microscope with an incidence angle between 51 and 53°. Objective was overlayed with immersion oil (Immersol^TM^ 518 F, ne = 1.518), and RIPK3_387–518_ fibrils were imaged via their autofluorescent properties using 473 nm laser line at < 10% power, sulfo-Cy5 labelled peptides were imaged using 640 nm laser line at < 5% power. Exposure set to 100 ms. Image processing for TIRF images was performed using FIJI/ImageJ analysis software. Background removal and beam correction were performed via image subtraction of the Gaussian blur (radius Σ = 80) of the TIRF image from the original.

### Single-molecule photobleaching

Cleaned glass coverslips (24 × 24 mm, 130–160 µm thickness, Epredia) were submerged in 100% acetone (~50 ml) in coplin jars. 2 ml silane (A3648) was added, and coplin jars were gently agitated to mix solution for 5 min then bath sonicated for 1 min. Coverslips were left to incubate in silane/acetone solution for a further 10 min then gently rinsed in MilliQ water and dried using N_2_ gas. Sulfo-Cy5 labelled A1*, A2* or A4* peptides (50–100 pM) were diluted out of DMSO into 50 mM Tris pH 8.0, 10 mM NaCl, then overlaid over coverslips which were mounted onto glass slides and sealed immediately with epoxy resin. Imaging was performed at TIRF angles using the 640 nm laser line at 6% power, with 100 ms exposure per frame until all detected fluorophores had bleached. Hyperstack images were analysed using the single molecule biophysics plugin on FIJI/ImageJ analysis software to identify molecule positions and measure photon count over time.

### Fluorescence assays using HT-29 cell models of cell death

HT-29 cells grown in TC treated T175 flasks (Corning^TM^) were aspirated, washed once with RT DPBS then trypsinised with 0.3 vol warmed trypsin EDTA for 3–5 min at 37 °C twice. Trypsin was quenched with 0.7 vol warmed D10 and then the mixture was centrifuged 400×g at RT for 5 min. Supernatant was discarded, then cells were resuspended in warm supplemented DMEM. A small sample was diluted 1:10 in trypan blue and loaded onto a haemocytometer for cell counting, then the cell suspension was diluted in warmed DMEM to 8.5 × 10^4^ cells/ml. Cells were seeded onto a 96-well clear bottom PhenoPlate (Revvity) coated in a collagen I matrix at 1.7 × 10^4^ cells/well and incubated at 37 °C in 5% CO_2_ overnight.

CellTracker^TM^ Orange CMRA (Invitrogen) was diluted to 5 μM in DMEM and warmed to 37 °C. HT-29 cells were washed once in DPBS, then CellTracker^TM^ Orange (50 μl) was added to each well (excluding wells to be incubated with Alexa Fluor 594-labelled control peptide) and incubated at 37 °C in 5% CO_2_ for 45 min. Following incubation, CellTracker^TM^ Orange solution was aspirated and replaced with 150 μl D10. Cells were treated to induce no cell death (0.125% DMSO vehicle), apoptosis (30 ng/μl hTNF + 1 μM BV6) or necroptosis (30 ng/μl hTNF + 1 μM BV6 + 25 μM zVADfmk), and then incubated at 37 °C in 5% CO_2_ for 6–7 h.

Treated and untreated cells were washed twice with 100 μl DPBS at RT. HT-29 cells were fixed with 4% PFA (100 μl) in the dark at RT for 25 min, then washed once with TBST before being permeabilised with ice cold absolute methanol (100 μl) in the dark at –30 °C for 10 min. Following fixation and permeabilisation steps, cells were washed twice, then stored in cold TBST, and plates were stored at 4 °C and protected from light overnight. Cells were blocked before the addition of peptide with 20% NDS solution diluted into TBST. 20% NDS solution (50 μl) was incubated with each well for 30 min at RT, then wells were washed twice in TBST before the addition of peptides.

Peptide master mixes were prepared in blocking buffer to 10 nM (10% final DMSO concentration) immediately prior to addition to wells. Peptide master mixes (50 μl) were added to each well and incubated in the dark at RT for 30 min, then washed twice with TBST. DAPI stain was prepared to 1 μg/ml in TBST and added (50 μl) to each well. Plates were incubated in the dark at RT for 30 min, then were sealed with optically clear sealing film and stored at 4 °C until imaging.

### Imaging and analysis of fluorescence

Imaging was performed on an Opera Phenix (PerkinElmer) at 63X magnification (water immersion) in confocal mode using the parameters given below. Z-stacking was performed at 1 µm intervals spanning 3 µm total distance. Image analysis was performed using Harmony 5.1 PhenoLOGIC analysis software (PerkinElmer). Intensity thresholds were adjusted as indicated to remove background fluorescence based on control wells for image exports. Analysis was performed on all whole wells. Cell counts were identified using DAPI stain (Method C) and cytoplasm identified by CellTracker^TM^ Orange stain (Method B), and all subsequent analysis was conducted on Cy5 fluorescence detected within cells as determined by these parameters. Total Cy5 intensity analysis, as well as spot analysis (spots detected using Method B), was performed measuring number of detected spots, mean spot size (px^2^) and spot fluorescence intensity (corrected and relative). Control peptide wells were excluded from image analysis due to the lack of cytoplasmic marker for cell threshold detection. Statistical significance between untreated, apoptosis and necroptosis induced conditions and between no peptide controls was determined by 2-way ANOVA testing using Tukey’s multiple comparisons tests.

**Table:** 

Dye	Excitation (nm)	Emission filter (nm)	Laser power (%)	Exposure (ms)
CellTracker Orange/ Alexa Fluor 594	561	570/50	100	100
DAPI	405	435/40	50	100
Alexa Fluor 488	488	500/40	100	100
Cy5	640	650/100	100	300

### Flow cytometry

HT-29 cells grown in TC-treated T75 flasks (Corning) were aspirated, washed once with RT DPBS then trypsinised with 0.4 vol warmed TrypLE^TM^ Express (Gibco) 3–5 min at RT. Trypsin was quenched with 0.8 vol cDMEM10 media, then cell suspension was centrifuged for 5 min 300*×*g at RT. Supernatant was discarded, then cells were resuspended in 0.8 vol cDMEM. Cell suspension was filtered through a 70 μm cell strainer, then a small aliquot was diluted 1:9 in trypan blue and loaded onto a haemocytometer for cell counting. Cells were diluted into warmed cDMEM to 0.1 × 10^6^ cells/ml, then 1 ml was seeded in duplicate for each condition (100 000 cells/well) onto a clear, TC-treated 12-well plate (Corning). Plate was incubated at 37 °C + 5% CO_2_ for 18–24 h.

Sulfo-Cy5-labelled peptides, free sulfo-Cy5 control or DMSO vehicle were incubated for 30 min at 250 nM in warmed serum-free Opti-MEM media. Cells were washed once with warmed serum-free Opti-MEM media, then peptide or control master mixes were added to a final concentration of 20 nM. Final DMSO concentration was 1%. Plates were incubated at 37 °C + 5% CO_2_ for 4 h. After incubation, media was aspirated and cells were trypsinised with 0.1 vol warmed TrypsinEDTA for 5 min at 37 °C. Trypsin was quenched with 0.1 vol warmed cDMEM10 media then cells were transferred to a 96-well V-bottom plate and centrifuged for 3 min at 350*×*g at RT. Supernatant was removed and cells were resuspended in an equal volume of PBS and centrifuged again for 3 min at 350*×*g at RT. A control sample was also prepared with a 1:1 suspension of untreated cells and cells that had been heated to 56 °C for 10 min Supernatant was removed, and 50 μl LIVE/DEAD™ Fixable Blue Stain (Invitrogen) (1:400 dilution in PBS) was added to cells. Cells were incubated for 30 min at RT, protected from light, then 100 μl PBS was added and cells were centrifuged for 3 min at 350*×*g at RT. Supernatant was removed, and cells were fixed by incubation of 100 μl 4% formaldehyde for 15–20 min at RT, protected from light. Cells were washed twice with 100 μl PBS and centrifugation for 5 min 500*×*g at RT. Cells were resuspended in 25 μl residual supernatant and transferred to 1.5 ml tubes. Wells were rinsed with 75 μl PBS to collect remaining cells, then samples stored on ice in the dark. Samples were taken for data acquisition on the LSR Fortessa (BD) flow cytometer, acquiring a minimum of 10 000 events for each sample.

### Confocal microscopy of fixed HT-29 cells with peptide added to live cells

Cells were prepared as described previously in preparation for flow cytometry. For confocal microscopy, cells were diluted to 5 × 10^4^ cells/ml. 200 μl was seeded in triplicate for each condition (10 000 cells/well) onto a black, optically clear flat bottom 96-well PhenoPlate (Revvity). Additional DPBS was added to the outer wells of the plate to minimise evaporation. Peptide and controls were added following the incubation period as described previously.

After 4-h incubation with peptides or controls, cells were washed with 200 μl serum-free Opti-MEM media then stained with 100 μl CellMask^TM^ Green plasma membrane stain (Invitrogen) (1:1000 dilution in Opti-MEM media) for 5–10 min at 37 °C. Cells were fixed in 100 μl warmed 4% formaldehyde at 37 °C for 5–10 min then washed thrice with 100 μl PBS. 50 μl Hoechst 33324 solution (Invitrogen) (1:400 dilution in PBS) was added to washed cells and incubated for 5 min at RT, then cells were washed thrice again in 100 μl PBS. Plates were stored at 4 °C protected from light and imaged within 24 h using an Opera Phenix (PerkinElmer) at 63X magnification (water immersion) in confocal mode with appropriate excitation/emission filters. Z-stacking was performed at 0.5 µm intervals spanning 7 µm total distance.

Images were used to calculate subcellular localisations of peptides within cells at the plasma membrane, within the cytoplasm or within the nucleus using the Harmony 5.1 PhenoLOGIC analysis software (PerkinElmer). Nuclei were identified by Hoechst staining, boundaries of the plasma membrane were defined by CellMask^TM^ Green staining, and the cytoplasm was classified as within the bounds between the nuclei and the cell membrane. Statistical significance between subcellular localisations for each peptide was determined by one-way ANOVA testing using Tukey’s multiple comparisons tests.

### 2D live-cell imaging of HT-29 cells following peptide incubation and TSV treatment

HT-29 cells were prepared in a 96-well black, optically clear flat bottom plate (Revvity) as described previously for confocal microscopy experiments. Peptides were preincubated with Opti-MEM media for 15 min at RT before being added to cells in media to a final concentration of 50 nM. Cells were incubated with peptides at 37 °C + 5% CO_2_ for 4 h. Following incubation, cells were washed once with 100 μl serum-free FluoroBrite™ DMEM media (FB-DMEM), then CellTracker™ Green CMFDA (1:400 dilution) and Hoechst 33342 (1:10 000 dilution) diluted in warmed FB-DMEM media was incubated with cells for 30 min at 37 °C + 5% CO_2._ After incubation, staining solution was removed and replaced with 100 μl warmed cFB-DMEM5 media supplemented with ProLong Live Antifade reagent (1:1000 dilution). Cells were treated with warmed TSZ solution (25 ng/ml hTNF, 1 μM BV-6, 25 μM zVAD-fmk) or vehicle (DMSO) in cFB-DMEM5 media containing 5 μg/ml propidium iodide. Plates were sealed with Breath-Easy^TM^ gas-permeable plate membrane (brand) then loaded into the Opera Phenix Plus (PerkinElmer) with environmental controls (37 °C + 5% CO_2_). Cells were imaged immediately using the 10X air objective (NA 0.3) and parameters given below. Images were acquired of 3–9 FOVs with 3 × 7.4 μm z-planes per well at 10–30 min intervals for 10 h.

**Table:** 

Dye	Excitation (nm)	Emission (nm)	Laser power (%)	Exposure (ms)
Hoechst 33342	361	497	75	100
CellTracker green	492	517	50	100
Propidium iodide	535	617	100	100
Cy5	647	665	100	100

## Supplementary material

online supplementary figure 1.

## Data Availability

All data will be made available upon request to the corresponding author.

## Open Access

Open access for this article was enabled by the participation of The University of Sydney in an all-inclusive Read & Publish agreement with Portland Press and the Biochemical Society.

## Abbreviations

RHIM: RIP homotypic interaction motif
RIPK3: receptor interacting protein kinase 3
RaPID: random non-standard peptide integrated discovery
ZBP1: Z-DNA-binding protein 1
