# Supplementary material for: De novo cyclic peptides allow visualisation of the monomeric and functional amyloid conformations of the kinase RIPK3
Source: Biochem J. 2026 Feb 4;483(2):191–210. doi: 10.1042/BCJ20250283 (PMC12905488; doi:10.1042/BCJ20250283)
Supplement: online supplementary figure 1. [file bcj-483-2-BCJ20250283-s001.docx]

Supplementary Figures and Table

[**Supplementary Figure 1:** ThT and TEM of BHUR3 2](#_Toc216699322)

[**Supplementary Figure 2:** ThT traces of RIPK3-binding peptides with and without BHUR3 3](#_Toc216699323)

[**Supplementary Figure 3:** TEM of A2 and A4 self-assembly with and without hRIPK3_387‑518_ 4](#_Toc216699324)

[**Supplementary Figure 4:** Single molecule photobleaching of A1*, A2* and A4* peptides 5](#_Toc216699325)

[**Supplementary Figure 5:** TEM of sulfo-Cy5-lablled tetra-lysine-tagged peptide self-assembly 6](#_Toc216699326)

[**Supplementary Figure 6:** Congo Red and turbidity data for A1*, A2* and A4* and tetra-lysine-tagged peptides 7](#_Toc216699327)

[**Supplementary Figure 7:** MST data of control amyloid proteins 8](#_Toc216699328)

[**Supplementary Figure 8:** Segmentation of 3D HT-29 cell images used for subcellular distribution analysis of sulfo-Cy5-labelled tetra‑lysine‑tagged peptides 9](#_Toc216699329)

[**Supplementary Table 1:** Summary of all calculated *K_d_* values from MST experiments 10](#_Toc216699330)

[**Supplementary Table 2:** Summary of total cells analysed for Figure 5B 11](#_Toc216699331)

[**Supplementary Table 3:** Summary of total cells analysed for Figure 6C 12](#_Toc216699332)


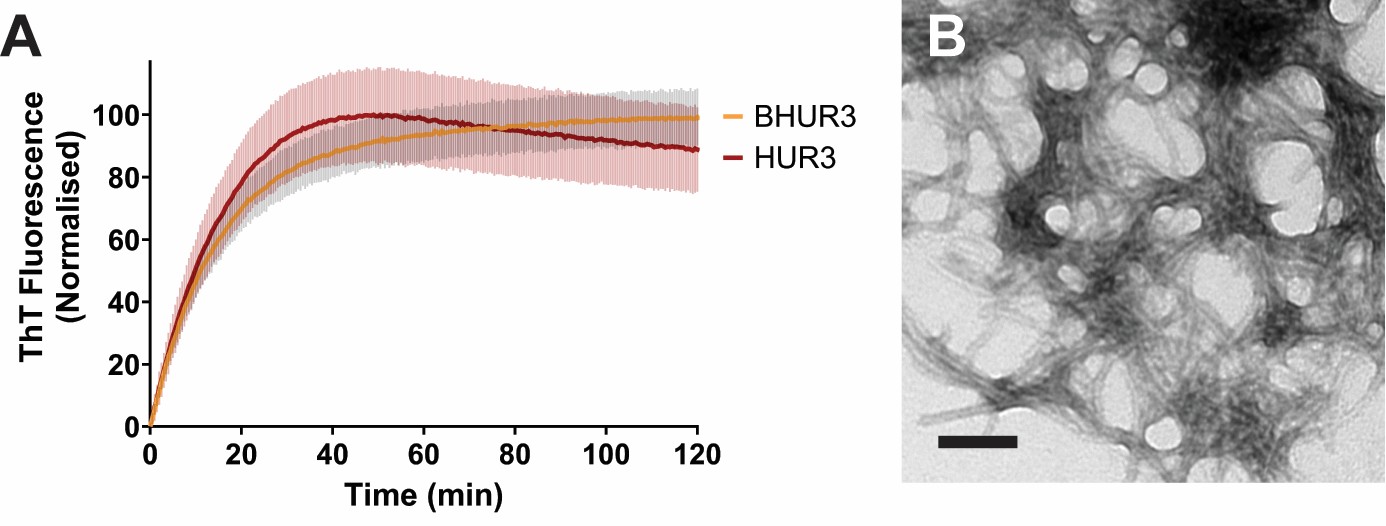


## **Supplementary Figure 1:** **ThT and TEM of BHUR3**

**.** A) ThT fluorescence from samples of 5 μM HUR3 or BHUR3 in PBS pH 7.4 at 37 ^o^C, normalised to plateau intensity. Error bars show SD. B) TEM of 0.2 mg/mL BHUR3 fibrils assembled in TBS, pH 8.0. Image taken at 110 000X magnification, scale bar represents 100 nm. Grid stained with 2% uranyl acetate.

**
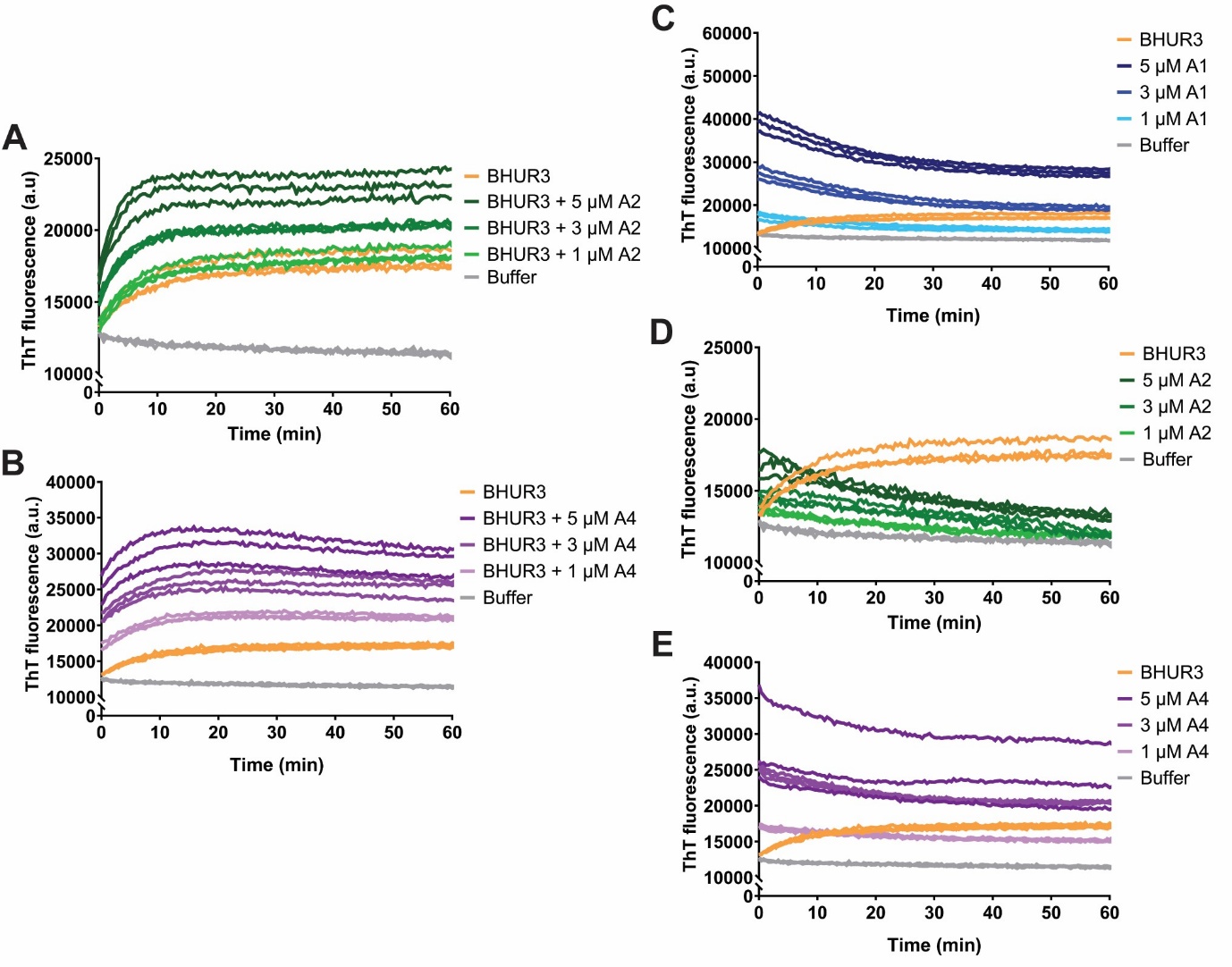
**

## **Supplementary Figure 2: ThT traces of RIPK3-binding peptides with and without BHUR3**

. Triplicate ThT traces of 1 μM BHUR3 co-incubated with 1, 3 or 5 μM A) A2, or B) A4. Triplicate ThT traces of 1 μM BHUR3 alone compared with 1, 3 or 5 μM C) A1, D) A2 or E) A4 alone. Assays performed at room temperature in PBS pH 7.4 supplemented with 40 μM ThT.

**
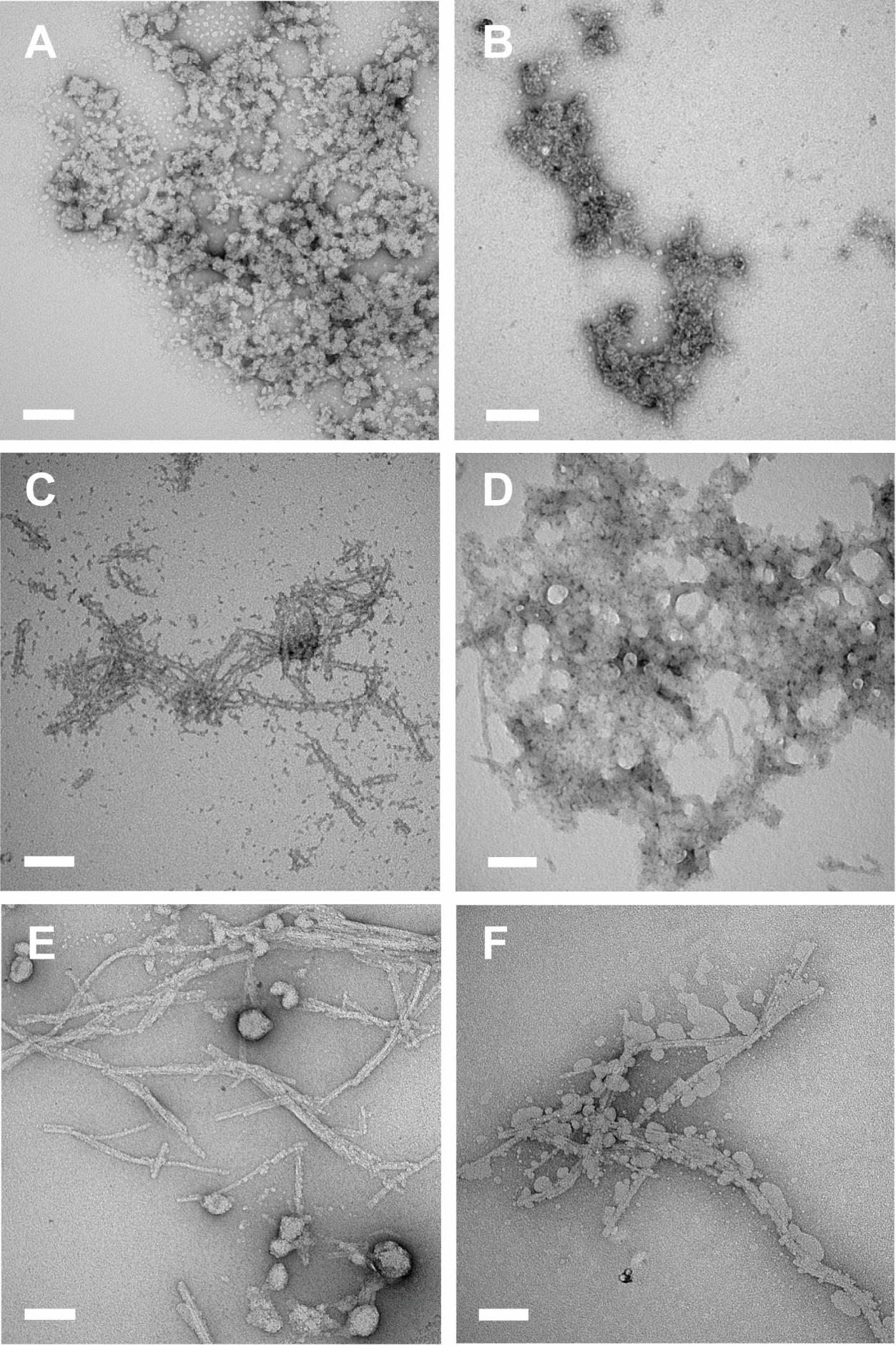
**

## **Supplementary Figure 3:** **TEM of A2 and A4 self-assembly with and without hRIPK3_387‑518_**

. TEM images of structures formed by A) 10 μM A2, and B) 10 μM A4 peptide alone. TEM images of 14 μM hRIPK3_387–518_ fibrils incubated with C) 2 μM A2, and D) 2 μM A4 peptide solutions. TEM images 14 μM insulin fibrils incubated with E) 2 μM A2, and F) 2 μM A4 peptide solutions. Images taken at 110 000X magnification, scale bars represent 100 nm. Grids were stained using 2% uranyl acetate.

**
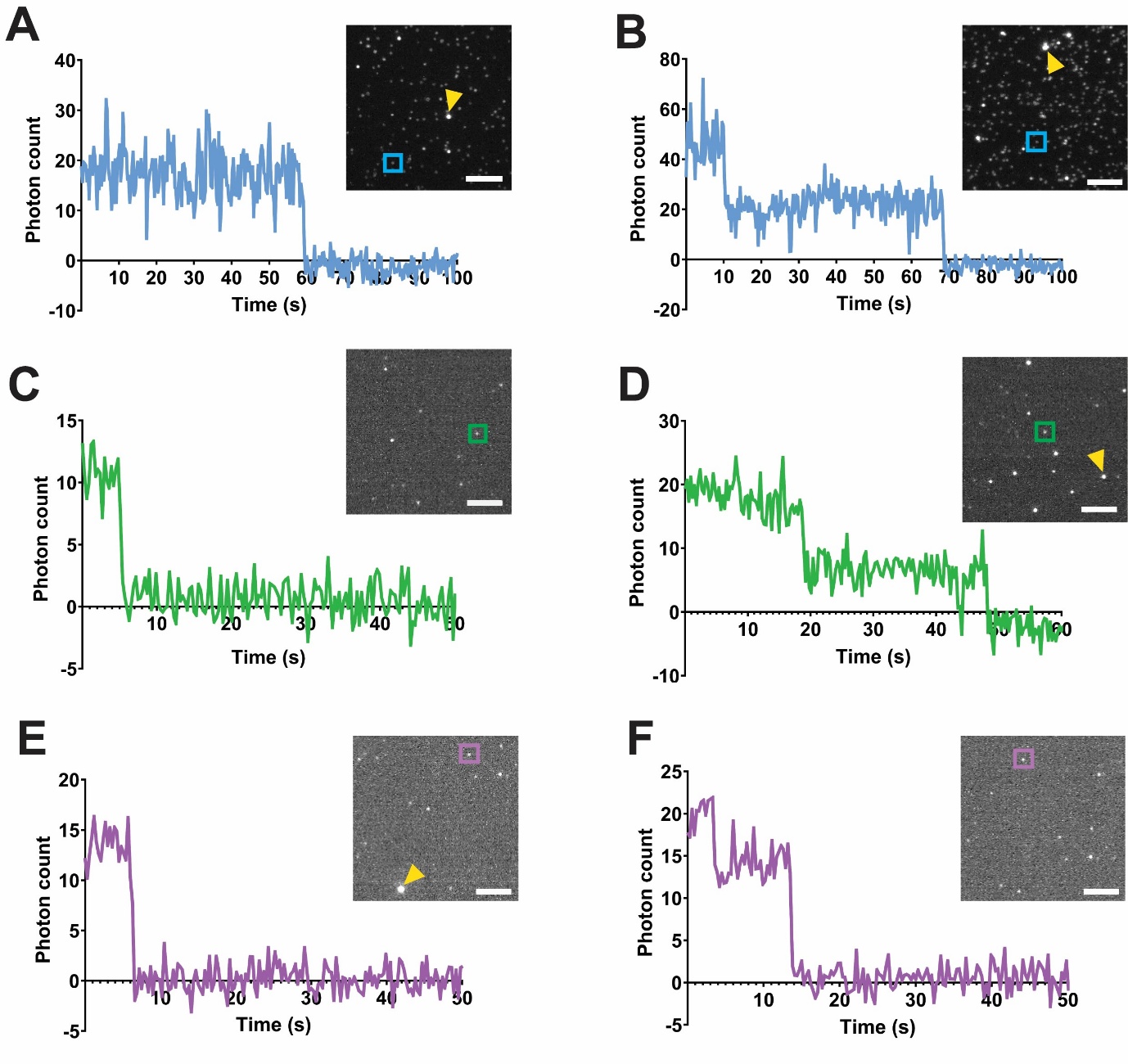
**

## **Supplementary Figure 4: Single molecule photobleaching of A1*, A2* and A4* peptides**

. Sulfo-Cy5-labelled A1* (50 pM) displaying A) monomeric, and B) dimeric species. Sulfo-Cy5-labelled A2* (50 pM) displaying C) monomeric, D) dimeric, and. Sulfo-Cy5-labelled A4* (100 pM) displaying E) monomeric, and F) dimeric. Coloured boxes indicate fluorescent spots corresponding to analysis shown. A1 in 50 mM Tris pH 8.0, 10 mM NaCl, 10% (w/v) D-glucose containing 1% GLOX solution (56 mg/mL glucose oxidase, 17 mg/mL catalyse, 10 mM Tris pH 8.0, 50 mM NaCl) and 10 mM MEA. A2 and A4 imaged without GLOX solution. Imaging performed in TIRF with 640 nm laser line (6% power), exposure at 300 ms/frame. Images corrected for background. Analysis performed using FIJI image analysis software. Images taken at 100X magnification, scale bars represent 5 μm. Examples of multimeric species with stoichiometry >4 indicated with yellow triangles.


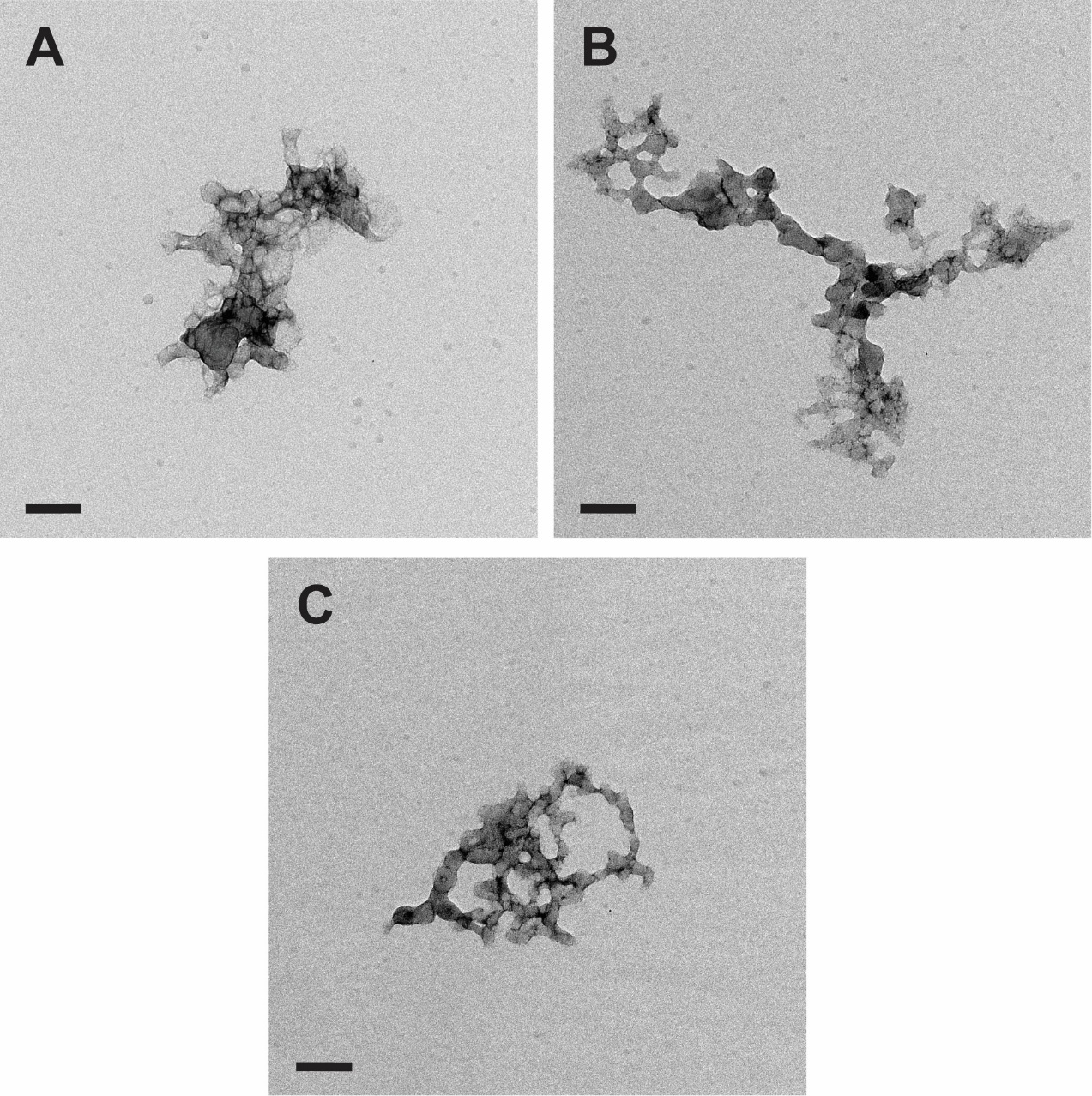


## **Supplementary Figure 5:** **TEM of sulfo-Cy5-lablled tetra-lysine-tagged peptide self-assembly**

. TEM images of structures formed by 2 μM A) A1Ktag, B) A2Ktag, or C) A4Ktag peptide alone. Images taken at 110 000X magnification, scale bars represent 100 nm. Grids were stained using 2% uranyl acetate.


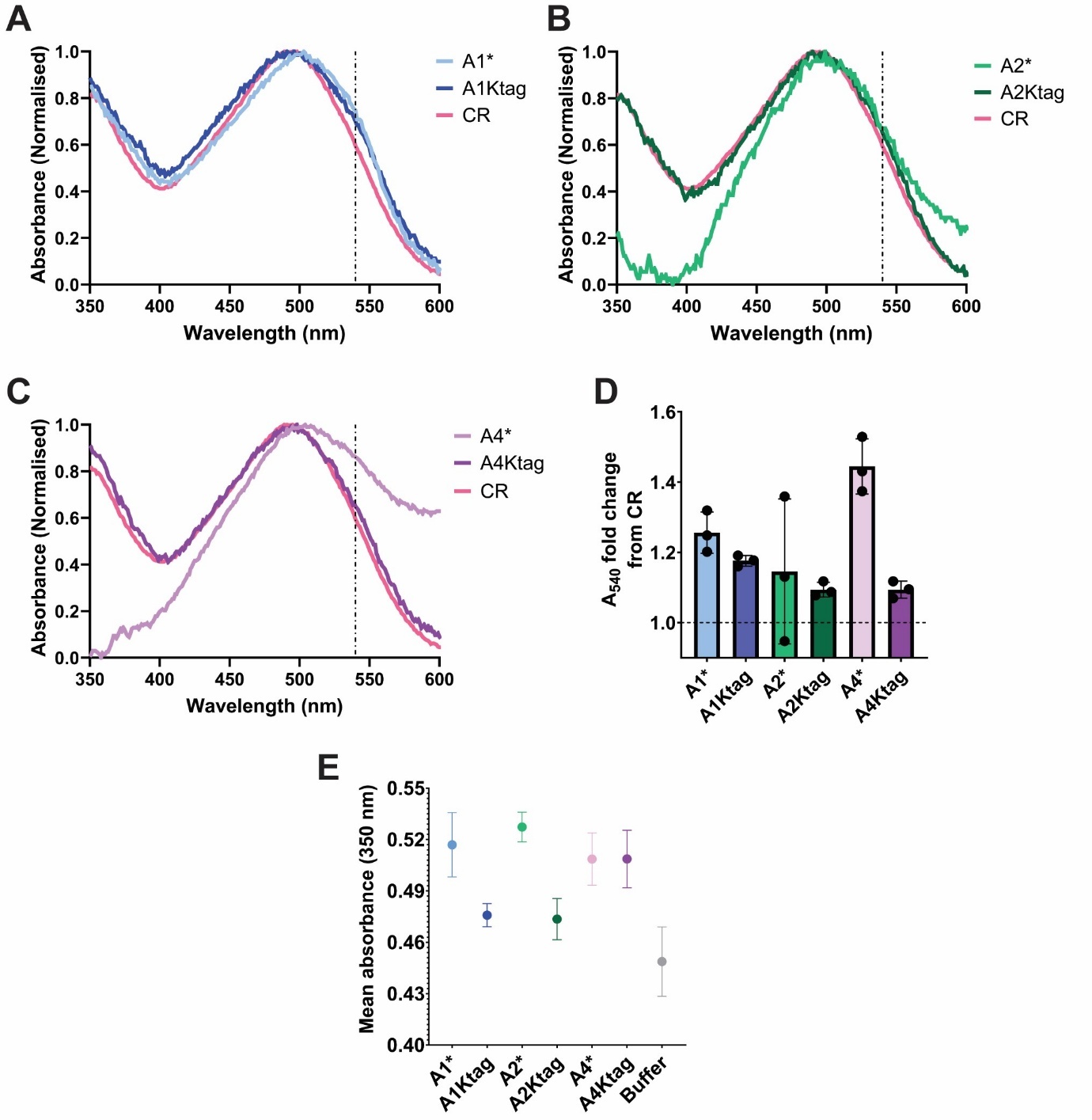


## **Supplementary Figure 6:** **Congo Red and turbidity data for A1*, A2* and A4* and tetra-lysine-tagged peptides**

. Normalised average absorbance of 1 μM Congo Red alone or in the presence of 0.15 mg/mL A) A1, B) A2, or C) A4 with and without a tetra‑lysine tag. Dotted line indicates 540 nm absorbance. D) Fold change of absorbance at 540 nm from CR alone control. Points indicate duplicate average of each experiment, n = 3. Error bars represent standard deviation. E) Turbidity as mean absorbance at 350 nm of A1*, A2* and A4* and their tetra‑lysine‑tagged counterparts compared with buffer (TBS‑T). Assay performed in triplicate, error bars indicate range.


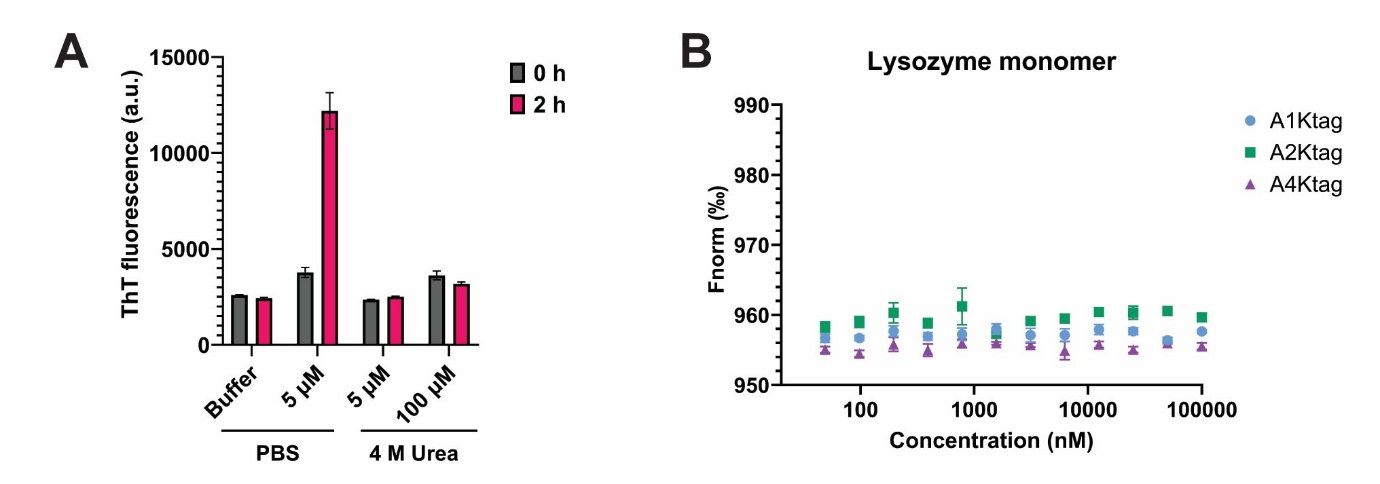


## **Supplementary Figure 7:** **MST data of control amyloid proteins**

. a) Mean initial (0 h) ThT fluorescence and endpoint fluorescence demonstrating monomeric nature of 5 μM and 100 μM HUR3 in 4 M urea over the course of 2 hours versus 5 μM HUR3 diluted into PBS. b) MST traces using 20 nM sulfo-Cy5-labelled tetra-lysine‑tagged peptides against lysozyme monomer. Raw data values shown as points with SD, fit values represented with solid line. Assays performed in triplicate n = 2. MST Assays were performed in TBS‑T pH 8.0, 0.1 mg/mL BSA, 0.04% glycerol, 5% DMSO.


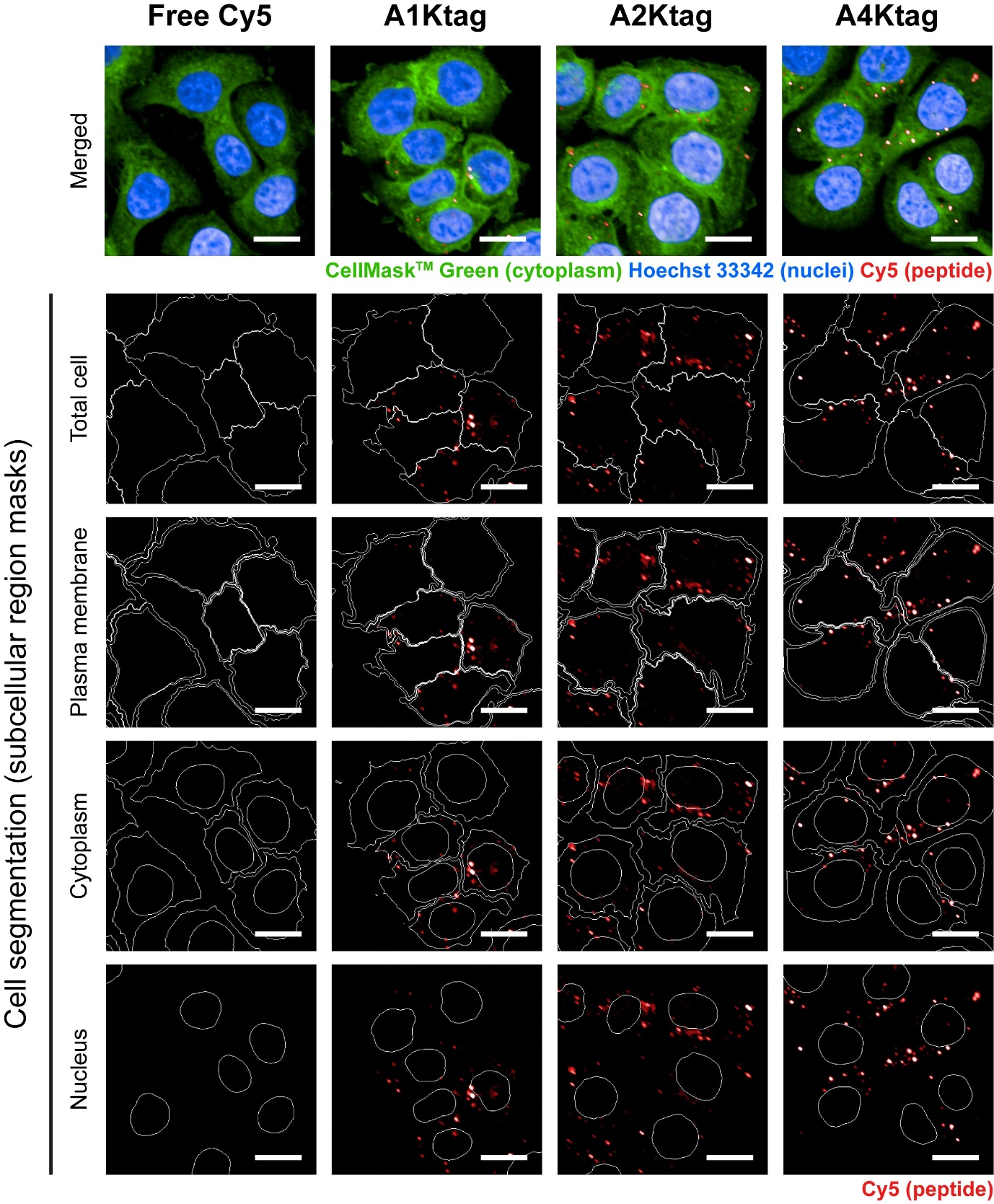


## **Supplementary Figure 8:** **Segmentation of 3D HT-29 cell images used for subcellular distribution analysis of sulfo-Cy5-labelled tetra‑lysine‑tagged peptides**

. Top: Maximum intensity projection of an example image showing 20 nM sulfo-Cy5 alone or sulfo-Cy5‑labelled A1Ktag, A2Ktag or A4Ktag peptide (red). Cells stained with Hoechst 33342 nuclear stain (blue) and CellMask^TM^ Green plasma membrane stain (green). Cell segmentation masks for total cell, plasma membrane, cytoplasm and nuclear regions of the cell utilised for analysis of subcellular distribution in Fig. 6C. Images taken at 63X magnification, scale bar represents 25 μm.

## **Supplementary Table 1:** Summary of all calculated *K_d_* values from MST experiments

| **Target** | **Peptide** | ***K_d_* (μM)** | ***K_d_* confidence (μM)** |
| --- | --- | --- | --- |
| RIPK3 fibril | A1Ktag | 1.96 | ± 0.27 |
|  | A2Ktag | 1.26 | ± 0.33 |
|  | A4Ktag | 2.30 | ± 0.39 |
| RIPK3 monomer | A1Ktag | 35.87 | ± 22.90 |
|  | A2Ktag | 45.52 | ± 19.25 |
|  | A4Ktag | 53.43 | ± 27.04 |
| IAPP fibril | A1Ktag | 49.02 | ± 9.77 |
|  | A2Ktag | 25.26 | ± 3.51 |
|  | A4Ktag | 24.80 | ± 4.80 |
| Insulin fibril | A1Ktag | 11.97 | ± 2.95 |
|  | A2Ktag | 6.10 | ± 1.14 |
|  | A4Ktag | 9.11 | ± 1.38 |

## **Supplementary Table 2:** Summary of total cells analysed for Figure 5B

| Condition | Peptide | Total no. cells analysed |
| --- | --- | --- |
| Untreated | A1Ktag | 2875 |
|  | A2Ktag | 3624 |
|  | A4Ktag | 3156 |
|  | No peptide | 5584 |
| Apoptosis | A1Ktag | 3966 |
|  | A2Ktag | 3804 |
|  | A4Ktag | 4680 |
|  | No peptide | 7307 |
| Necroptosis | A1Ktag | 5887 |
|  | A2Ktag | 6164 |
|  | A4Ktag | 4583 |
|  | No peptide | 10552 |

## **Supplementary Table 3:** Summary of total cells analysed for Figure 6C

| Independent experiment | Peptide | Total no. cells analysed |
| --- | --- | --- |
| Replicate 1 | A1Ktag | 817 |
|  | A2Ktag | 804 |
|  | A4Ktag | 851 |
|  | Sulfo-Cy5 | 805 |
|  | DMSO | 755 |
| Replicate 2 | A1Ktag | 545 |
|  | A2Ktag | 525 |
|  | A4Ktag | 500 |
|  | Sulfo-Cy5 | 712 |
|  | DMSO | 686 |
| Replicate 3 | A1Ktag | 1224 |
|  | A2Ktag | 1371 |
|  | A4Ktag | 1325 |
|  | Sulfo-Cy5 | 1551 |
|  | DMSO | 1483 |
| Total | A1Ktag | 2586 |
|  | A2Ktag | 2700 |
|  | A4Ktag | 2676 |
|  | Sulfo-Cy5 | 3068 |
|  | DMSO | 2924 |
